# Pharmacologic MRI Brain Imaging Studies of Serotonin 5‐HT_1_ Receptor Agonists in Awake Mice

**DOI:** 10.1002/prp2.70199

**Published:** 2026-04-23

**Authors:** Brittany M. Brems, Erin E. Sullivan, Ryan P. McGlynn, Praveen Kulkarni, Craig F. Ferris, Raymond G. Booth

**Affiliations:** ^1^ Center for Drug Discovery Northeastern University Boston Massachusetts USA; ^2^ Department of Pharmaceutical Sciences Northeastern University Boston Massachusetts USA; ^3^ Department of Chemistry and Chemical Biology Northeastern University Boston Massachusetts USA; ^4^ Center for Translational Neuroimaging Northeastern University Boston Massachusetts USA; ^5^ Department of Psychology Northeastern University Boston Massachusetts USA

**Keywords:** aminotetralin, awake animal imaging, BOLD, FPT, NLX‐112, phMRI, serotonin receptor

## Abstract

Serotonin (5‐hydroxytryptamine, 5‐HT) type‐1 G protein‐coupled receptors are expressed throughout the central nervous system. 5‐HT_1A_R activation is the putative mechanism of approved drugs for generalized anxiety disorder and major depressive disorder and is being studied in the treatment of autism and neurological disorders. The 5‐HT_1B_ and 5‐HT_1D_Rs are the putative therapeutic targets for “triptan”‐type migraine drugs, and the 5‐HT_1B_R is associated with prosocial effects, relative to autism treatment, consistent with its high expression in limbic and cortical brain regions. Under study is a recently developed drug candidate for autism, (*S*)‐5‐(2′‐fluorophenyl)‐2‐dimethylaminotetralin (FPT), that is a full efficacy pan‐5‐HT_1_R agonist (pEC50 = 7.4, 9.4, and 8.6 at 5‐HT1_A_, 5‐HT1_B_, and 5‐HT1_D_Rs, respectively). FPT demonstrates anti‐seizure, anxiolytic, and prosocial properties, as well as reduces stereotypic movements in *Fmr1* knockout mice, a model for autism. The goal of this study was to compare brain activation patterns of the pan‐5‐HT_1_R agonist FPT to NLX‐112, a highly selective 5‐HT_1A_R full agonist (pEC_50_ = 7.5) which also prevents seizures in *Fmr1* knockout mice, to help establish therapeutic mechanisms in autism. We used pharmacological magnetic resonance imaging (phMRI) in awake C57BL/J6 mice to assess activation of integrated neuronal circuits as measured by blood oxygen level dependent volume of activation changes, comparing dose‐related effects of FPT and NLX‐112. The selective 5HT_1A_R agonist NLX‐112 broadly inhibited brain activity in a dose‐dependent manner. In contrast, FPT increased global brain activity; however, dose‐related effects were complex, suggesting FPT's polypharmacology at 5‐HT_1_Rs and perhaps other receptors are involved in its brain activation pattern.

**Abbreviations**
5‐HT: serotonin, 5‐hydroxytryptamine
5‐HT_1A_R: serotonin 1a receptor
5‐HT_1B_R: serotonin 1b receptor
5‐HT_1D_R: serotonin 1d receptor
5‐HT_1E_R: serotonin 1e receptor
5‐HT_1F_R: serotonin 1f receptor
5‐HT_2A_R: serotonin 2a receptor
5‐HT_7_R: serotonin 7 receptor
5‐SAT: 5‐substituted‐2‐aminotetralin
ADHD: attention deficit hyperactivity disorder
BOLD: blood oxygen level dependent
CNS: central nervous system
ctx: cortex
D_1_R: dopamine D1 receptor
D_2_R: dopamine D2 receptor
FDR: false discovery rate
FPT: (S)‐5‐(2′‐fluorophenyl)‐2‐dimethylaminotetralin
GPCR: G‐protein coupled receptor
HASTE: Half Fourier Acquisition Single Shot Turbo Spin Echo
KO: knockout
LID: l‐DOPA induced dyskinesia
n.: nucleus
NLX‐112: (3‐Chloro‐4‐fluorophenyl‐[4‐fluoro‐4‐([(5‐methylpyridin‐2‐yl)methylamino]methyl)piperidin‐1‐yl]methanone)
phMRI: pharmacological magnetic resonance imaging
RARE: rapid acquisition relaxation enhancement
ROI: region of interest
S.C.: subcutaneous
SE: standard error
VOA: volume of activation
α_1_R: adrenergic 1 receptor
α_2_aR: adrenergic 2a receptor
α_2_cR: adrenergic 2c receptor

## 1 Introduction

The serotonin (5‐hydroxytryptamine, 5‐HT) 1 receptor family consists of five subtypes (5‐HT_1A_ , _1B_ , _1D_ , _1E_ , _1F_ ) which are G‐protein coupled receptors (GPCRs) that canonically couple to a G_α_i protein to inhibit adenylyl cyclase activity [1]. The 5‐HT_1_R subtypes are expressed in peripheral tissues and have a dense and broad distribution in the brain [2], where they play a role in neuropsychiatric disorders, such an anxiety [3], schizophrenia [4], and substance use disorder [5], as well as, neurological disorders, including pain [5, 6] and Parkinson's disease [4, 7].

The 5‐HT_1A_R subtype, which is expressed throughout the cortex, hippocampus, amygdala, and raphe nuclei [8, 9], is the putative therapeutic target of the drugs buspirone [10] and gepirone [11], approved for generalized anxiety disorder and major depressive disorder, respectively. Buspirone and gepirone are partial agonists at the 5‐HT_1A_R and have other activities at aminergic receptors [12, 13] associated with the parent drug and metabolites [14, 15].

The 5‐HT_1B_R is expressed in the frontal cortex, basal ganglia, striatum, and hippocampus and has been shown to have prosocial and anti‐aggression properties upon activation [16]. The 5‐HT_1D_R is expressed in the cortex, caudate‐putamen, and dorsal raphe nuclei [17]. The “triptan”‐type migraine headache drugs are agonists at the 5‐HT_1B_ and 5‐HT_1D_Rs.

The 5‐HT_1E_R is expressed in the cortex, amygdala, and caudate putamen [18, 19]; however, characterization of the physiological roles in humans has been hampered since mice and rats lack the 5‐HT_1E_ R gene [20]. The 5‐HT_1F_R is expressed in the hypothalamus, locus coeruleus, and trigeminal ganglia [21], where it regulates neuropathic pain and neuroinflammation [22], and is the putative therapeutic target of the “ditan”‐type migraine drugs [23, 24].

**FIGURE 1:**
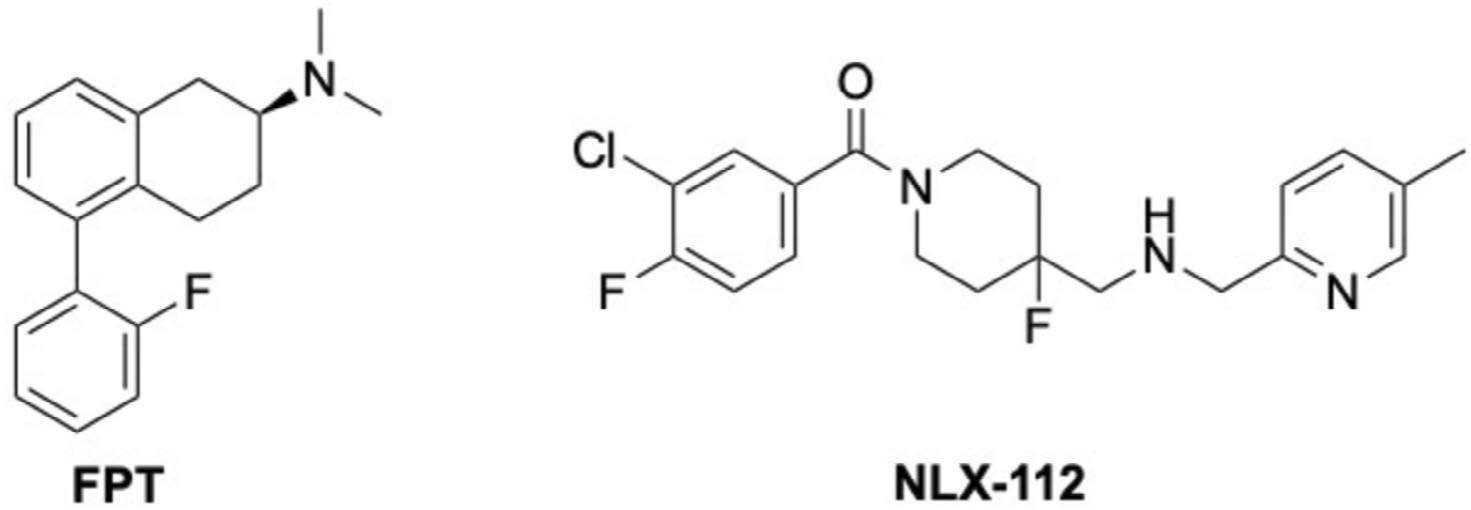
Chemical structures of FPT and NLX‐112.

Recently, we developed a new 5‐HT_1_R agonist based on a novel 5‐substituted‐2‐aminotetralin (5‐SAT) chemotype [25], that is, (*S*)‐5‐(2′‐fluorophenyl)‐2‐dimethylaminotetralin, hereon FPT (Figure 1). FPT is a full‐efficacy 5‐HT_1_R agonist that has equipotent activity at the 5‐HT_1B_ (pEC50 = 9.4) and 5‐HT_1D_Rs (pEC50 = 9) and is ~100 times less potent (pEC50 = 7.4) at the 5‐HT_1A_R, with nil 5‐HT_1F_R activity (5‐HT_1E_R not assessed) [26]. FPT also has partial agonist activity at the 5‐HT_7_R (pEC_50_ = 7.5) [27] and the α_2A_R (pEC_50_ = 9.3) [28], and partial inverse agonist activity at the α_2C_R (pIC_50_ = 7.1) [28], but nil activity at the 5‐HT_2A_ and dopamine D_2_Rs (pEC_50_ < 5) [27]. FPT has been shown to completely prevent audiogenic‐induced seizures in *Fmr1* knockout (KO) mice, a model for the most common monogenetic form of autism (fragile X syndrome), without impacting locomotor behavior [29]. In tests for anxiolytic activity, FPT decreased marble‐burying, repetitive grooming, and rearing, and increased social approach behavior [29]. Notably, about 25% of autism patients (aged 13 and older) have comorbid seizures [30, 31] and up to 84% have anxiety disorders [32, 33], along with the diagnostic criteria of deficits in social communications and interactions [34]. There also is considerable evidence in the literature that activation of 5‐HT_1_Rs can elicit an antiepileptic effect in a variety of seizure types [35].

In comparison to FPT, NLX‐112 (3‐chloro‐4‐fluorophenyl‐[4‐fluoro‐4‐([(5‐methylpyridin‐2‐yl)methylamino]methyl)piperidin‐1‐yl]methanone) (Figure 1) is a long‐known [5] selective 5‐HT_1A_R full efficacy agonist (pEC_50_ = 7.5), with nil activity at 5‐HT_1B_, 5‐HT_1D_, 5‐HT_1F_Rs [26]. NLX‐112 also has no appreciable affinity at 5‐HT_2_ , α_1_‐ and α_2_ , and D_1_ and D_2_Rs [12]. Like FPT, NLX‐112 was reported to prevent audiogenic‐induced seizures in *Fmr1* KO mice [36]—however, in those studies, NLX‐112 caused motor impairment including hypolocomotion, flat body posture, backward walking, head weaving, and orofacial movements that are characteristic of other selective 5‐HT_1A_R agonists such as (R)‐8‐OH‐DPAT, and which were not observed with FPT. Despite its negative effects on motor function in *Fmr1* KO mice, NLX‐112 recently underwent a Phase 2a clinical trial for l‐DOPA induced dyskinesia (LID) in Parkinson's disease wherein safety and efficacy readouts were established [37].

With the goal of establishing neurotherapeutic mechanisms in the treatment of autism symptoms, including seizures, we compared dose‐related effects of the pan‐5‐HT_1A/1B/1D_R agonist FPT and the 5‐HT_1A_ selective agonist NLX‐112 using pharmacological magnetic resonance imaging (phMRI) in awake mice. We assessed activation of integrated neuronal circuits as measured by blood oxygen level‐dependent volume of activation (BOLD VOA) [38], as well as global brain activation, taking into account the polypharmacology of FPT (and perhaps the unknown activities of NLX‐112).

## 2 Methods

### 2.1 Animal Usage

Male C57BL/J6 mice (*n* = 50) approximately 50 days of age and weighing between 20 and 30 g were obtained from Charles River Laboratories (Wilmington, Massachusetts, USA). Mice were kept on a 12 h light–dark cycle with 5 mice to a cage and *ad libitum* access to food and water. The mice were acquired and cared for in accordance with the guidelines outlined in the “Guide for the Care and Use of Laboratory Animals” (National Institutes of Health Publications No. 85‐23, Revised 1985), and adhered to the National Institutes of Health and the American Association for Laboratory Animal Science guidelines. The protocols used in this study complied with the regulations of the Institutional Animal Care and Use Committee at Northeastern University protocol 23‐0406R and complied with the ARRIVE guidelines for reporting in vivo experiments in animal research [39]. Animals were monitored daily over the duration of the study for general health, food, and water consumption. A 15% loss in body weight was set as a humane endpoint.

### 2.2 Drug Preparation and Administration

FPT was synthesized as previously described [25, 28]. NLX‐112 was purchased from Sigma Aldrich. Both were dissolved in 0.9% NaCl for S.C. injections. Ten mice each were randomly assigned to vehicle or one of two compound groups corresponding to FPT or NLX‐112, and received only that compound throughout the study, at three different doses: 0.03, 0.3, or 3.0 mg/kg; there was a minimum 3‐day washout period between doses. Doses were chosen based on previous research indicating a subcutaneous (S.C.) injection of 3.0 mg/kg FPT in mice reaches a brain concentration of 5 μM after 30 min, which is sufficient to alleviate repetitive behaviors (stereotypy), a diagnostic feature of autism [27]; comparable doses (0.04 and 0.16 mg/kg, intraperitoneally) of NLX‐112 are reported to reverse l‐DOPA‐induced abnormal involuntary movements in hemiparkinsonian rats with established dyskinesia [40]. To deliver the drug remotely during the imaging session, a polyethylene tube (PE‐20), approximately 30 cm in length, was positioned subcutaneously. Each day of imaging had a mix of drug and drug doses known by all the investigators. Blinding was not necessary for this study since BOLD VOA was the objective physiological readout exclusively used in our analyses. Additionally, there is sufficient automation in key analysis steps to mitigate bias.

### 2.3 Awake Mouse Imaging

#### 2.3.1 Imaging System

A detailed description of the awake mouse imaging system is published elsewhere [41]. Notably, we used a quadrature transmit‐receive volume coil (ID = 38 mm) that provided both high anatomical resolution and high signal‐to‐noise ratio for voxel‐based BOLD fMRI. The unique design of the mouse holder (Ekam Imaging, Boston MA) fully stabilizes the mouse head in a cushioned helmet, minimizing discomfort caused by ear bars and other common restraint systems used for immobilization during awake animal imaging [42]. Mice with motion exceeding 100 μm during the scan, that is greater than one‐half the in‐plane dimensions of a voxel (ca 190 μm^2^) in any orthogonal direction were excluded from the study. No mice were excluded during acclimation. Based on these criteria the final number of mice in each experimental condition was vehicle (*n* = 10), FPT 0.03 mg/kg (*n* = 8), FPT 0.3 mg/kg (*n* = 8), FPT 3.0 mg/kg (*n* = 10), NLX‐112 0.03 mg/kg (*n* = 8), NLX‐112 0.3 mg/kg (*n* = 8), and NLX‐112 3.0 mg/kg (*n* = 8) with 10 mice originally assigned to each group.

#### 2.3.2 Acclimation

Mice were acclimated to the restraining system and noise produced by the scanner 1 week prior to imaging. Briefly, the mice were anesthetized with 1%–2% isoflurane and secured into the head holder. Once consciousness was regained, the mice were placed in an enclosed dark box simulating the MRI scanner (emitting audio recordings of MRI pulses). The process of acclimation was repeated for 5 consecutive days: the first session lasting 20 min, the second session for 30 min, and the following three for 45 min. This process of acclimation is done in order to reduce the effects caused by the autonomic nervous system during awake animal imaging (changes in heart rate, respiration, corticosteroid levels, motor movements, etc.) in order to obtain overall improved image quality [43].

#### 2.3.3 BOLD phMRI Image Acquisition and Pulse Sequence

Experiments were conducted using a Bruker BioSpec 7.0/20‐cm USR horizontal magnet (Bruker) and a 2 T/m magnetic field gradient insert (ID = 12 cm) capable of 120‐μs rise time. Beginning each imaging session, a high‐resolution anatomical data set was collected using the rapid acquisition relaxation enhancement (RARE) pulse sequence (18 slices; 0.75 mm; FOV 1.8 cm^2^; data matrix 128 × 128; TR 2.1 s; TE 12.4 ms; effect TE 48 ms; NEX 6; 6.5 min acquisition time). Functional images were acquired using a multi‐slice Half Fourier Acquisition Single Shot Turbo Spin Echo (HASTE) pulse sequence (18 slices; 0.75 mm; FOV 1.8 cm^2^; data matrix 96 × 96; TR 6 s; TE 4 ms; effect TE 24 ms; 35 min acquisition time; in‐plane resolution 187.5 μm^2^). The use of spin echo was crucial in obtaining functional images that possessed the anatomical fidelity required for aligning the data with the mouse MRI atlas, as shown in previous publications [41]. Each functional imaging session consisted of uninterrupted data acquisitions (whole brain scans) of 350 scan repetitions or acquisitions for a total elapsed time of 35 min. The control window included the first 50 scan acquisitions covering a 5 min baseline. Following the control window, a S.C. injection of vehicle, FPT, or NLX‐112 was given followed by another 300 acquisitions over a 30 min period. The order of drug doses was randomized over the scanning sessions.

#### 2.3.4 Imaging Data Analysis

The dose‐dependent effects of FPT and NLX‐112 on brain activity were quantified through positive and negative percent changes in BOLD signal relative to baseline. The initial analyses of signal change in individual subjects were done by comparing image acquisitions 300–345 to baseline 5–50. The statistical significance of these alterations was evaluated for each voxel (approximately 15 000 per subject) in their original reference system using independent Student's *t*‐tests. A threshold of 1% was employed to accommodate the typical fluctuations in the BOLD signal observed in the awake rodent brain. To address the issue of multiple *t*‐tests conducted, we used the following formula to control the false‐positive detection rate below 0.05:

$$P_{i}\leq \frac{i}{V}\frac{q}{c\left(V\right)},$$

In our analysis, the formula was used to control false positives involved evaluating the *p*‐values (*P*_*i*_) obtained from the *t*‐tests for each pixel within the region of interest (ROI). The ROI contained a total of *V* pixels, and these pixels were ranked based on their probability values. To maintain conservative estimates of significance, a false‐positive filter value (*q*) of 0.2 was applied. Additionally, *c*(*V*) was set to unity, following the approach outlined by Benjamini and Hochberg [44]. The resulting statistical significance was determined based on a 95% confidence level, two‐tailed distributions, and the assumption of heteroscedastic variance for the *t*‐tests. Pixels that displayed statistical significance retained their relative percentage change values, while all other pixel values were assigned a value of zero. Voxel‐based percent changes in BOLD signal generated for individual subjects were combined across subjects within the same group to build representative functional maps. To this end, all images were first aligned and registered to a 3D Mouse Brain Atlas with 135 segmented and annotated brain regions (Ekam Solutions). The co‐registrational code SPM8 was used with the following parameters: Quality 0.97, Smoothing 0.4 mm, Separation 0.6 mm. Gaussian smoothing was performed with a FWHM of 0.5 mm. Image registration involved translation, rotation, and scaling, independently and in all three dimensions. All applied spatial transformations were compiled into a matrix [*T*_*j*_]^−1^ for the *j*th subject. Every transformed anatomical pixel location was tagged with a brain area to generate fully segmented representations of individual subjects within the atlas. Next, composite maps of the percent changes in BOLD signal were built for each experimental group. Each composite pixel location (row, column, and slice) was mapped to a voxel of the *j*th subject by virtue of the inverse transformation matrix [*T*_*j*_]^−1^. A tri‐linear interpolation of subject‐specific voxel values determined their contribution to the composite representation. The use of the inverse matrices ensured that the full composite volume was populated with subject inputs. The average of all contributions was assigned as the percent change in BOLD signal at each voxel within the compositive representation of the brain for the respective experimental group. The number of activated voxels in each of the 135 regions was then compared between the vehicle and the respective drug (FPT, NLX‐112) doses using a Kruskal‐Wallis test statistic. Those brain areas with significant changes in positive and negative BOLD signal are presented in Tables 1 and 2 for FPT at the 3.0 mg/kg dose, and the data for all 135 brain areas for both positive and negative BOLD of FPT is presented in Tables S1a and S1b. Those brain areas with significant changes in positive and negative BOLD signal are presented in Tables 3 and 4 for NLX‐112 at the 3.0 mg/kg dose, and the data for all 135 brain areas for both positive and negative BOLD of NLX‐112 is presented in Tables S2a and S2b. Each measure for the four experimental groups was compared with a one‐way ANOVA followed by Tukey's multiple comparison test using GraphPad Prism version 9.1.2 for Windows (GraphPad Software).

**TABLE 1:** Positive volume of activation Veh versus 3.0 mg/kg FPT.

Brain area	Vehicle			FPT		*p*	Ω Sq
	Mean	SE		Mean	SE		
3rd cerebellar lobule	0	0.0	<	42	7.6	0.000	0.771
5th cerebellar lobule	1	0.4	<	81	15.0	0.000	0.012
6th cerebellar lobule	3	1.4	<	121	24.8	0.000	0.714
Accumbens shell	1	0.6	<	21	4.8	0.000	0.698
Fimbria hippocampus	13	3.0	<	41	4.6	0.000	0.672
Tenia tecta ctx	4	2.1	<	28	3.0	0.000	0.644
7th cerebellar lobule	0	0.1	<	27	8.1	0.001	0.605
Accumbens core	1	0.9	<	11	4.0	0.001	0.604
Medullary reticular dorsal n.	56	18.7	>	7	2.1	0.001	0.589
Orbital ctx	11	3.9	<	74	24.6	0.001	0.540
Frontal association ctx	2	0.6	<	32	13.7	0.002	0.519
Prelimbic ctx	2	1.5	<	24	8.2	0.002	0.518
Simple lobule cerebellum	24	11.3	<	91	10.6	0.002	0.000
Ventral pallidum	5	2.5	<	23	7.0	0.002	0.014
Stria terminalis	18	6.0	>	0	0.1	0.002	0.477
Paramedian lobule	13	2.9	<	58	10.8	0.003	0.463
Vestibular n.	10	4.6	<	55	14.8	0.003	0.450
Principal sensory n. trigeminal	13	6.3	<	55	11.9	0.003	0.008
Granular cell layer	5	1.5	<	34	6.9	0.003	0.435
Ambiguus n.	2	1.1	<	7	1.5	0.003	0.435
Anterior thalamic n.	5	2.0	<	19	4.4	0.005	0.397
8th cerebellar lobule	54	11.9	>	16	10.5	0.006	0.368
Locus coeruleus	0	0.0	<	1	0.5	0.007	0.036
Lateral rostral hypothalamic n.	22	4.1	<	51	10.4	0.009	0.328
Lateral paragigantocellular n.	17	4.6	<	39	6.0	0.010	0.029
Diagonal band of Broca	3	1.1	<	12	3.3	0.011	0.033
Anterior pretectal thalamic n.	5	1.7	<	12	2.3	0.011	0.032
Anterior commissure	0	0.3	<	3	1.7	0.012	0.302
Lateral dorsal thalamic n.	5	1.5	<	15	3.8	0.014	0.023
Medial preoptic n.	9	3.1	<	27	6.2	0.015	0.280
Secondary motor ctx	31	11.0	<	78	12.9	0.015	0.023
Medial mammillary n.	6	2.8	<	19	3.3	0.016	0.005
Prepositus n.	0	0.1	<	6	2.3	0.017	0.267
Medullary reticular ventral n.	56	19.1	<	16	5.5	0.018	0.013
Crus of ansiform lobule	118	33.2	<	228	32.0	0.019	0.021
10th cerebellar lobule	0	0.1	<	13	5.7	0.019	0.027
Lateral lemniscus	4	2.6	<	12	2.8	0.020	0.023
Anterior amygdaloid n.	2	0.7	<	10	2.4	0.020	0.040
Pyramidal tracts	5	2.0	<	18	5.9	0.022	0.032
Endopiriform n.	0	0.2	<	4	1.6	0.022	0.240
Pituitary	4	2.0	<	14	4.4	0.023	0.238
Cerebral peduncle	31	12.1	<	88	25.0	0.026	0.041
Flocculus cerebellum	37	12.0	<	80	15.7	0.026	0.036
Retrosplenial rostral ctx	42	14.5	<	79	10.1	0.032	0.022
Dorsal hippocampal commissure	4	1.6	<	9	1.5	0.035	0.195
Lateral reticular n.	2	2.3	<	8	2.9	0.036	0.192
Inferior colliculus	89	26.0	<	179	27.3	0.039	0.036
Lemniscal n.	6	3.4	<	19	5.4	0.042	0.034
Anterior cingulate n.	15	4.3	<	37	8.2	0.047	0.166

**TABLE 2:** Negative volume of activation Veh versus 3.0 mg/kg FPT.

Brain area	Vehicle			FPT		*p*	Ω Sq
	Mean	SE		Mean	SE		
Medullary reticular dorsal n.	38	11.8	>	6	2.5	0.001	0.604
Bed n. stria terminalis	16	5.2	>	2	1.6	0.002	0.437
Lateral rostral hypothalamic n.	14	4.7	>	2	0.8	0.003	0.421
Ambiguus n.	70	23.6	>	6	1.7	0.004	0.410
Glomerular layer	162	26.4	>	66	8.6	0.005	0.360
Lateral septal n.	48	8.0	>	12	7.8	0.007	0.334
Caudate putamen	286	38.8	>	128	41.1	0.008	0.318
Spinal trigeminal n.	12	4.6	<	61	17.3	0.009	0.308
Lateral preoptic n.	2	1.4	>	0	0.0	0.011	0.232
Frontal association ctx	62	10.3	>	25	6.1	0.012	0.319
Stria terminalis	6	4.7	>	0	0.1	0.012	0.309
Dorsal medial hypothalamic n.	4	1.5	>	1	0.4	0.013	0.271
Granular cell layer	113	21.0	>	55	9.3	0.013	0.301
Medial septal n.	4	0.9	>	2	1.3	0.018	0.248
Infralimbic ctx	3	0.9	>	1	1.0	0.018	0.248
Fimbria hippocampus	25	6.2	>	9	5.0	0.025	0.256
Corpus callosum	49	11.3	>	16	6.1	0.025	0.215
Lateral reticular n.	31	7.4	>	10	3.4	0.028	0.221
Anterior cingulate n.	40	9.5	>	18	10.3	0.029	0.211
Orbital ctx	104	22.4	>	47	17.0	0.032	0.226
Optic tract	2	0.9	>	0	0.1	0.038	0.142
Anterior olfactory n.	102	16.0	>	60	11.0	0.039	0.172
Endopiriform n.	15	3.2	>	9	1.5	0.043	0.185
Fornix	1	0.4	>	0	0.3	0.047	0.179
Dorsal hippocampal commissure	2	1.1	>	0	0.2	0.049	0.133

**TABLE 3:** Positive volume of activation Veh versus 3.0 mg/kg NLX‐112.

Brain area	Vehicle			NLX‐112		*p*	Ω Sq
	Mean	SE		Mean	SE		
3rd cerebellar lobule	0	0.0	<	14	2.9	0.000	0.844
Accumbens core	1	0.9	<	19	3.7	0.000	0.797
Accumbens shell	1	0.6	<	23	3.6	0.001	0.011
6th cerebellar lobule	3	1.4	<	136	13.0	0.001	0.736
Anterior commissure	0	0.3	<	5	0.9	0.001	0.717
Pyramidal tracts	5	2.0	<	18	1.6	0.001	0.689
Corpus callosum	38	7.9	<	80	5.9	0.002	0.624
Medullary reticular dorsal n.	56	18.7	>	7	1.9	0.002	0.624
Tenia tecta ctx	4	2.1	<	19	2.8	0.002	0.612
8th cerebellar lobule	54	11.9	>	6	3.3	0.002	0.025
Posterior thalamic n.	8	3.7	>	0	0.0	0.004	0.512
Bed n. stria terminalis	3	1.5	<	17	4.5	0.006	0.004
10th cerebellar lobule	0	0.1	<	7	2.6	0.008	0.420
Lateral rostral hypothalamic n.	22	4.1	<	52	7.3	0.009	0.406
Caudal piriform ctx	76	10.3	>	41	4.2	0.009	0.406
Medullary reticular ventral n.	56	19.1	>	15	4.3	0.012	0.037
Ambiguus n.	2	1.1	<	5	1.1	0.013	0.352
2nd cerebellar lobule	13	3.3	<	26	2.8	0.013	0.350
5th cerebellar lobule	1	0.4	<	9	2.5	0.014	0.350
Endopiriform n.	0	0.2	<	7	1.9	0.014	0.350
Stria terminalis	18	6.0	>	0	0.3	0.014	0.350
Granular cell layer	5	1.5	<	28	6.2	0.015	0.334
Orbital ctx	11	3.9	<	47	11.3	0.015	0.333
Habenular n.	3	0.9	>	1	0.9	0.016	0.331
Ventral tegmental n.	3	1.4	<	12	2.5	0.017	0.320
Anterior amygdaloid n.	2	0.7	<	8	1.7	0.017	0.319
Fimbria hippocampus	13	3.0	<	25	3.0	0.024	0.281
Principal sensory n. trigeminal	13	6.3	<	33	3.7	0.024	0.281
7th cerebellar lobule	0	0.1	<	19	6.9	0.026	0.272
Anterior thalamic n.	5	2.0	<	12	1.2	0.029	0.024
Ventral pallidum	5	2.5	<	24	6.0	0.031	0.250
Medial preoptic n.	9	3.1	<	24	5.7	0.031	0.249
Lateral preoptic n.	2	0.9	<	6	1.4	0.033	0.018
9th cerebellar lobule	17	4.6	>	5	1.5	0.035	0.234
Cortical amygdaloid n.	53	7.7	>	31	4.1	0.035	0.039
Diagonal band of Broca	3	1.1	<	10	2.9	0.039	0.222
Dorsal hippocampal commissure	4	1.6	>	0	0.4	0.040	0.042
Frontal association ctx	2	0.6	<	13	3.7	0.042	0.213
Lateral lemniscus	4	2.6	<	11	1.9	0.043	0.211
Anterior olfactory n.	24	5.3	<	39	7.0	0.046	0.203
Lateral reticular n.	2	2.3	<	5	1.5	0.049	0.195

**TABLE 4:** Negative volume of activation Veh versus 3.0 mg/kg NLX‐112.

Brain area	Vehicle			NLX‐112		*p*	Ω Sq
	Mean	SE		Mean	SE		
Prepositus n.	2	0.9	<	16	1.1	0.001	0.759
5th cerebellar lobule	3	1.3	<	64	11.7	0.001	0.710
Intermediate reticular n.	7	3.7	<	49	6.8	0.001	0.650
Gigantocelllaris reticular n.	16	4.9	<	147	16.5	0.001	0.654
Paramedian lobule	10	4.1	<	69	13.6	0.001	0.577
Spinal trigeminal nuclear n.	12	4.6	<	72	10.5	0.002	0.580
Medullary reticular dorsal n.	38	11.8	>	6	1.3	0.002	0.624
Vestibular n.	12	7.2	<	78	14.1	0.002	0.515
Parabrachial n.	4	1.9	<	17	1.9	0.002	0.536
Pontine reticular n. caudal	35	14.8	<	130	10.7	0.002	0.518
10th cerebellar lobule	2	0.9	<	16	3.4	0.003	0.547
Parvicellular reticular n.	14	3.8	<	36	3.1	0.003	0.473
Lateral geniculate	2	1.3	<	15	3.4	0.003	0.483
7th cerebellar lobule	2	0.9	<	31	13.6	0.004	0.488
Cerebral peduncle	32	12.5	<	103	13.4	0.005	0.442
Temporal ctx	24	4.8	>	6	2.8	0.006	0.421
2nd cerebellar lobule	2	1.1	<	23	5.5	0.007	0.424
Reticulotegmental n.	13	5.6	<	35	2.3	0.007	0.446
Cerebellar nuclear n.	3	1.1	<	23	5.4	0.007	0.398
Interpeduncular n.	5	2.5	<	15	2.6	0.008	0.380
9th cerebellar lobule	5	1.7	<	36	8.7	0.008	0.393
Glomerular layer	162	26.4	>	63	14.1	0.009	0.365
Olivary complex	4	2.0	<	13	2.9	0.010	0.366
Pedunculopontine tegmental n.	12	6.0	<	37	4.7	0.010	0.358
Lateral rostral hypothalamic n.	14	4.7	<	37	5.5	0.010	0.388
Lateral posterior thalamic n.	7	3.6	<	21	1.8	0.010	0.383
Subiculum	38	27.0	<	78	11.0	0.016	0.308
Posterior hypothalamic n.	2	0.9	<	7	1.2	0.016	0.300
Lateral paragigantocellular n.	5	1.7	<	15	2.8	0.018	0.291
Ambiguus n.	70	23.6	>	7	1.2	0.018	0.322
Anterior pretectal thalamic n.	8	3.6	<	20	2.3	0.020	0.287
Ventral pallidum	14	5.1	<	32	5.4	0.021	0.294
Retrosplenial caudal ctx	27	16.6	<	54	11.9	0.021	0.280
Superior colliculus	56	32.2	<	140	15.6	0.021	0.277
Median raphe n.	16	6.9	<	39	3.6	0.023	0.263
Lateral caudal hypothalamic n.	7	2.8	<	19	3.7	0.026	0.242
Pontine n.	28	12.6	<	72	8.7	0.027	0.252
Posterior thalamic n.	8	4.1	<	22	3.5	0.029	0.287
Lateral reticular n.	31	7.4	>	8	2.9	0.035	0.241
Dentate gyrus	54	25.8	<	125	10.9	0.036	0.209
Secondary somaotsensory ctx	46	17.4	<	86	9.3	0.036	0.217
Dorsal medial hypothalamic n.	4	1.5	<	9	1.5	0.044	0.213
Caudal piriform ctx	48	8.2	<	78	11.1	0.046	0.219

### 2.4 Nomenclature of Targets and Ligands

Key protein targets and ligands in this article are hyperlinked to corresponding entries in http://www.guidetopharmacology.org, the common portal for data from the IUPHAR/BPS Guide to PHARMACOLOGY [45], and are permanently archived in the Concise Guide to PHARMACOLOGY 2019/20 [46].

## 3 Results

**FIGURE 2:**
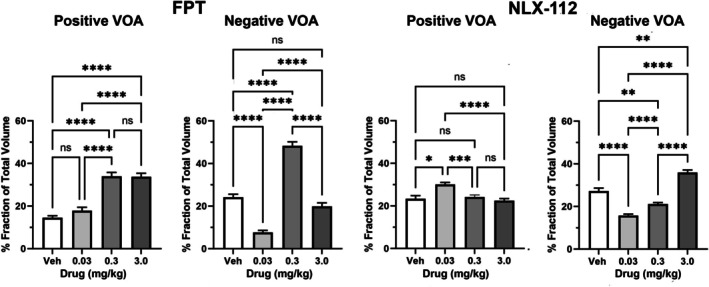
Dose‐dependent changes in positive and negative BOLD volume of activation (VOA) for FPT and NLX‐112 as % fraction of the total number of voxels in a brain. ns *p* > 0.05, *p < 0.05, **p < 0.01, ***p < 0.001, ****p < 0.0001.

Figure 2 shows the dose‐dependent changes in positive and negative BOLD volume of activation (VOA) for FPT and NLX‐112 as % fraction of the total number of voxels in subject brain. The bar graphs for vehicle, 0.03, 0.3, and 3.0 mg/kg doses represent the mean ± SE for all brain areas in the mouse MRI atlas. A matched one‐way ANOVA showed a significant treatment effect for all conditions. A Tukey's multiple comparison post hoc test was used to compare the doses within each treatment group. For FPT, there is an increase in positive BOLD VOA (*F*_(2.41, 248.2)_ = 33.72, *p* < 0.0001), at both the 0.3 and 3.0 mg/kg doses greater than vehicle. The negative BOLD VOA for FPT (*F*_(2.31, 245.5)_ = 95.5, *p* < 0.0001) appears as an inverted U‐shape dose–response relationship with the intermediate dose of 0.3 mg/kg being significantly higher than the 0.03 and 3.0 mg/kg doses as well as vehicle. However, it must be noted that the Figure 2 graph shows global brain activation and inactivation—when analyzed by brain region, there were only 7 regions (out of 135) that showed a significant increase in negative BOLD VOA at the FPT 0.3 mg/kg dose compared to vehicle (Table S1b). Thus, the large increase in negative BOLD VOA at the 0.3 mg/kg dose of FPT can be due to the accumulation of signal from many brain regions which each, on their own, did not show a significant change in BOLD VOA from vehicle, but when added together, yielded a significant effect. This can account for the discrepancy for ~90% global brain activation or inactivation, while other doses do not exceed 60% global brain activation or inactivation.

For NLX‐112, the lowest dose (0.03 mg/kg) produces a positive BOLD VOA (*F*_(2.30, 308.9)_ = 6.988, *p* = 0.0006) that is significantly greater than all other treatments, as well as vehicle. Also, there is a dose‐dependent decrease in positive VOA between the 0.3 and 3.0 mg/kg doses compared to the 0.03 mg/kg dose. In contrast, there is a dose‐dependent increase in negative BOLD VOA for NLX‐112 (*F*_(1.91, 256.2)_ = 50.35, *p* < 0.0001) where each dose is different from each other and vehicle. However, the lowest dose of NLX‐112 shows less negative BOLD VOA compared to vehicle.

**FIGURE 3:**
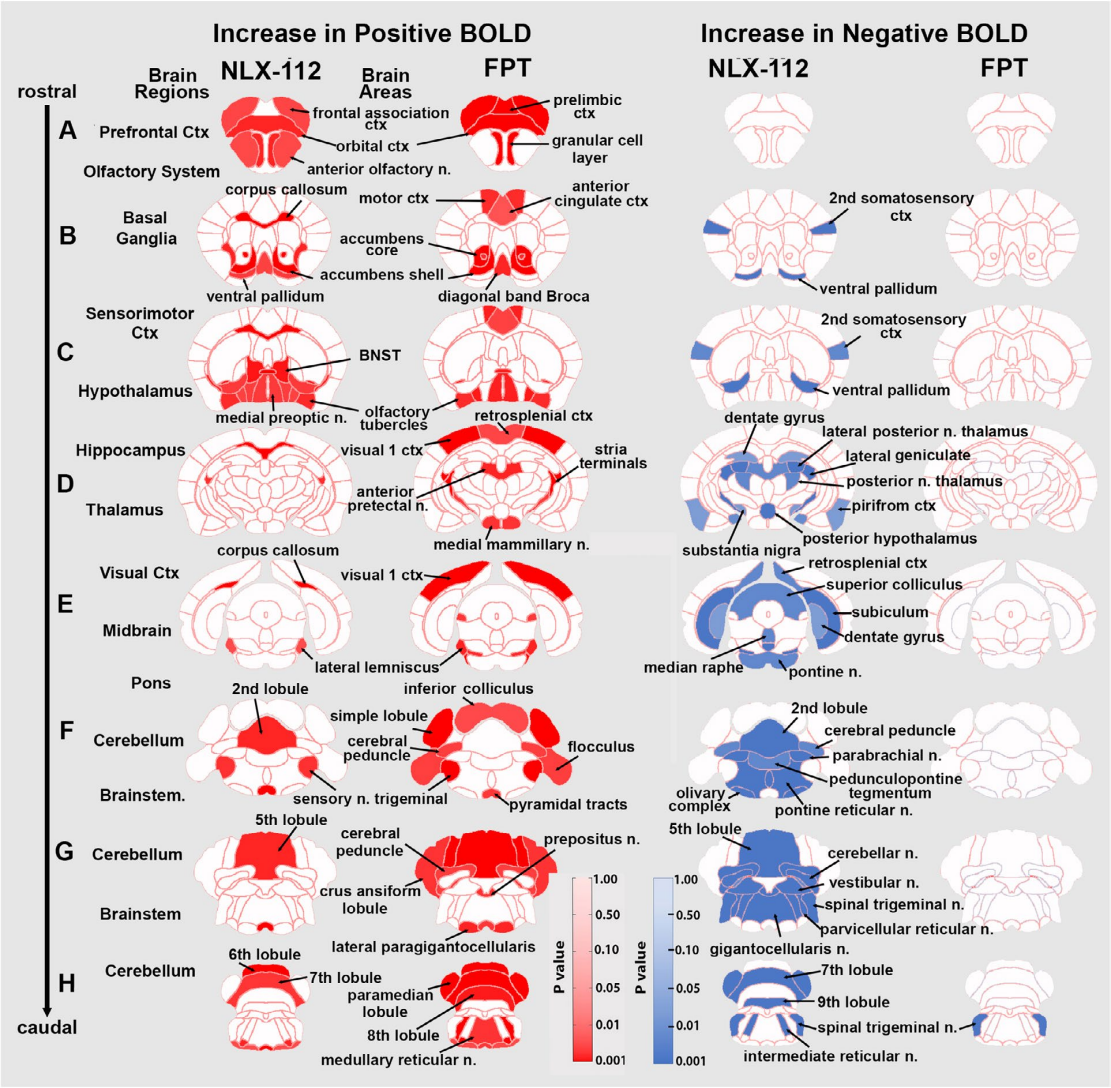
FPT and NLX‐112 statistical heat maps. 2D coronal sections show the localization of brain areas that were significantly different between vehicle and the 3.0 mg/kg dose of FPT and NLX‐112 in positive (left) and negative (right) BOLD signaling. Sections are aligned rostral (top) to caudal (bottom).

Based on the observed brain‐wide dose‐dependent changes in VOA, we chose to compare the 3.0 mg/kg doses of FPT and NLX‐112. Table 1 is a truncated list of 49/135 brain areas, all of which showed a significant increase in the positive BOLD VOA in response to the 3.0 mg/kg dose FPT, compared to vehicle. Brain areas are ranked in order of their significance using a critical value of *p* < 0.05. Shown are the mean ± SE together with the *p*‐value and effect size as omega square (Ω Sq). A false discovery rate (FDR) for multi‐comparisons gives a significance level of *p* = 0.060. In all cases but three, (highlighted in gray) FPT increased VOA. The most sensitive areas are localized to the back of the brain in the cerebellum and the forebrain for example, accumbens, orbital, prelimbic, and frontal association cortices (see Figure 3). Table 2 is a truncated list of 27/135 brain areas, all of which showed a significant decrease in VOA as compared to vehicle, except for spinal trigeminal n. (highlighted in gray); the FDR is *p* = 0.039. Several of these areas (e.g., orbital, frontal association and anterior cingulate cortices) are listed in Table 1, which emphasizes that increases in positive BOLD VOA generally are accompanied by decreases in negative BOLD at other voxels in the same brain area.

Table 3 is a truncated list of 42/135 brain areas, all of which showed a positive BOLD VOA in response to the 3.0 mg/kg dose of NLX‐112, except for the 10 brain areas highlighted in gray. The most sensitive areas were localized to the cerebellum and forebrain. Table 4 lists 44/135 brain areas, all of which showed an increase in negative VOA in response to NLX‐112 as compared to vehicle, except for the five brain areas highlighted in gray.

Figure 3 summarizes the data from Tables 1, 2, 3, 4 as statistical heat maps showing the location of the brain areas affected by the 3.0 mg/kg dose of FPT and NLX‐112. The coronal sections are labeled (A—H) and arranged from rostral (top) to caudal (bottom). Areas in red denote a significant increase in positive BOLD VOA while areas in blue denote a significant increase in negative BOLD VOA. NLX‐112 is active mainly in the front of the brain, while FPT is active throughout the brain with the only increase in negative BOLD seen in the spinal trigeminal n. (section H). In brain section (A), representing the prefrontal cortex (ctx) and olfactory system, there is an increase in positive BOLD VOA for NLX‐112 and FPT throughout the region, with the exception of a lack of activation in the anterior olfactory n. by FPT. Notably, there is no negative BOLD VOA for either drug in the prefrontal cortex and olfactory region. Brain section (B) highlights the basal ganglia, where activation is observed in the accumbens and ventral pallidum, but not in the striatum. The ventral pallidum shows an increase in both positive and negative BOLD VOA for NLX‐112. Section (C) highlights the sensitivity of the hypothalamus to NLX‐112, as well as the absence of any activity (positive or negative BOLD VOA) in the sensorimotor cortex for either drug. Section (D) shows the hippocampus and thalamus where neither drug has a positive effect, while NLX‐112 increases negative BOLD in the dentate gyrus and several dorsal thalamic nuclei. Section (E) highlights more of the hippocampus together with the visual cortex (ctx), pons, and midbrain. FPT increases activity in the visual ctx while NLX‐112 increases negative BOLD VOA in the hippocampus and median raphe. Sections (F—H) highlight the many areas of the cerebellum and brainstem. The distinction between FPT and NLX‐112 is most apparent in these hindbrain areas. FPT activates most of the cerebellum while NLX‐112 is confined to the 5th, 6th, and 7th lobules. In contrast, NLX‐112 has a pronounced effect on increasing negative BOLD VOA in the cerebellum, including in the deep cerebellar nuclei and much of the brainstem that includes several areas that comprise the ascending reticular activating system (e.g., pontine reticular n., parabrachial n., parvicellular reticular n., and gigantocellularis n.) This region also consists of the only brain region, the spinal trigeminal n., that exhibits an increase in negative BOLD VOA from FPT.

**FIGURE 4:**
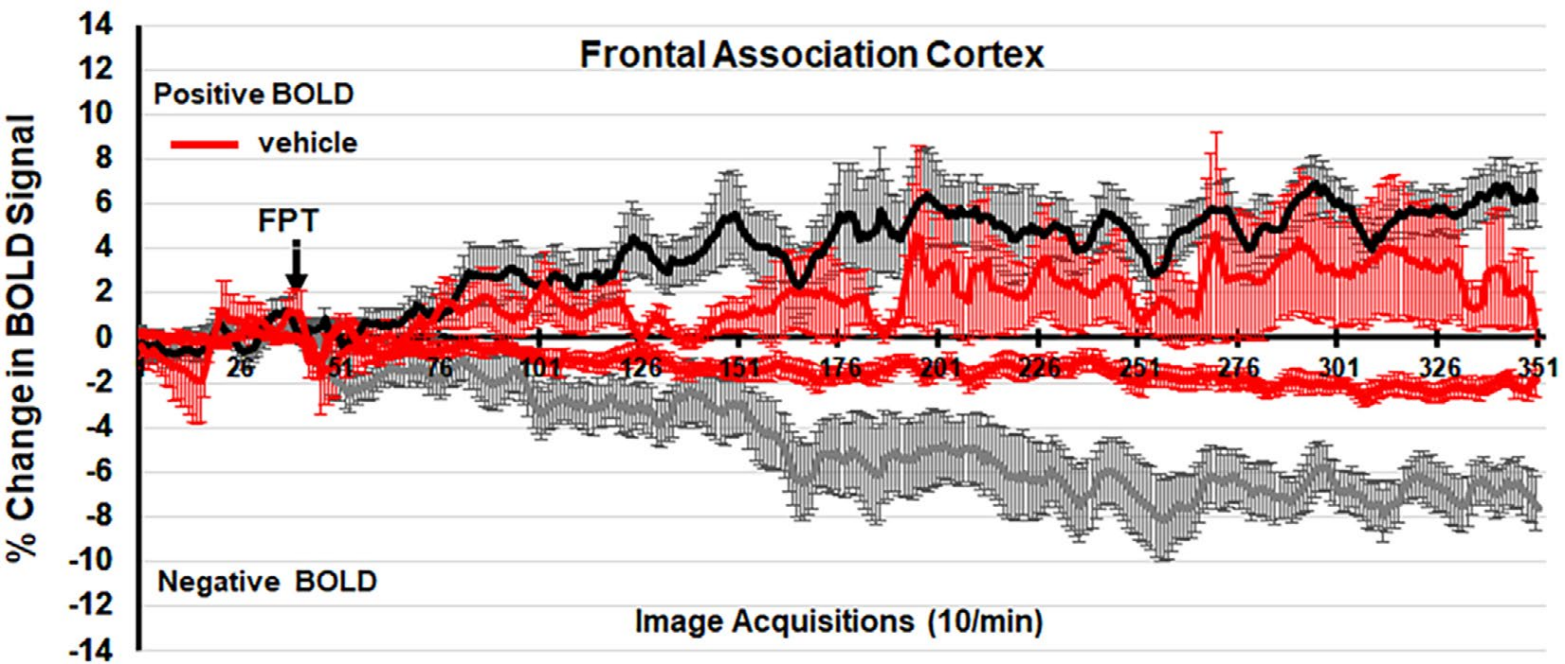
Percent positive and negative BOLD change over time at 3 mg/kg FPT in the frontal association cortex. The 1% threshold represents the level of background noise typical in awake animal imaging.

**FIGURE 5:**
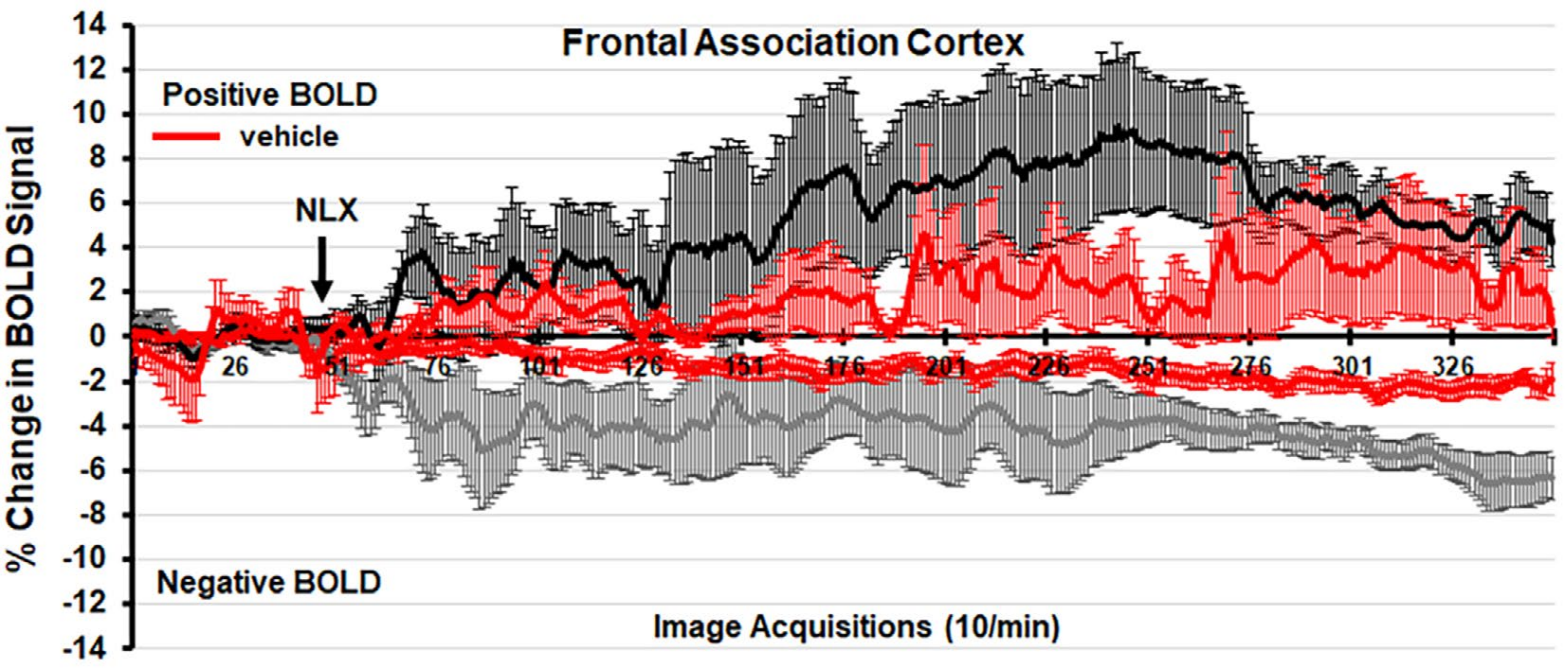
Percent positive and negative BOLD change over time at 3 mg/kg NLX‐112 in the frontal association cortex. The 1% threshold represents the level of background noise typical in awake animal imaging.

Figures 4 and 5 show the percent change in BOLD signal over time following injection (arrow) of vehicle or 3 mg/kg dose of FPT and NLX‐112, respectively, occurring at approximately the 50th image acquisition (5 min from onset of imaging). The mean ± SE for each of the 350‐image acquisitions is the composite of the frontal association cortex areas listed in Tables 1, 2, 3, 4. Possible signal drift does not affect the interpretation of net drug response seen in Tables 1, 2, 3, 4 since BOLD VOA at 300 to 345‐image acquisitions is exclusively compared.

## 4 Discussion

Previous imaging studies have been done using NLX‐112 [40, 47, 48], however, the current study is unique because it was performed in awake mice, without anesthesia, and used higher doses of up to 3.0 mg/kg. NLX‐112 is reportedly highly selective for the 5‐HT_1A_R over other 5‐HT_1_R subtypes and other receptor systems, thus serving as an excellent comparator for compounds such as FPT that have activity at several 5‐HT_1_ subtypes, 5‐HT_7_, and adrenergic α_2_ GPCRs. Previous in vivo studies have shown that FPT has anxiolytic, prosocial, and antiepileptic effects and it is hypothesized that this polypharmacology may be beneficial in eliciting these effects [29].

The present study showed that global brain activity was increased by FPT, albeit dose‐related effects were complex, consistent with FPT's complex pharmacology. The FPT 0.03 mg/kg dose significantly reduced negative BOLD VOA while the 0.3 and 3.0 mg/kg doses significantly increased positive BOLD VOA compared to vehicle. The dose–response for FPT regarding negative BOLD VOA was biphasic, as the reduced signal at 0.03 mg/kg changed to a significantly increased signal compared to vehicle (and other FPT doses) at 0.3 mg/kg, and at 3.0 mg/kg. The FPT signal was not significantly different from vehicle (but was significantly different from the 0.03 and 0.3 mg/kg doses). As noted, only 7 out of 135 brain regions had a significant increase in negative BOLD VOA at the 0.3 mg/kg dose of FPT, suggesting that the accumulation of signal from many regions, which individually did not have significant changes compared to vehicle, showed a significant combined effect. In contrast to FPT, NLX‐112 inhibited global brain activity in a typical dose‐dependent manner, as indicated by a significantly increased negative BOLD VOA at each of the doses tested (0.03, 0.3, 3.0 mg/kg) compared to vehicle. A dose–response relationship was not apparent for NLX‐112 regarding positive BOLD VOA, as the 0.03 mg/kg dose produced a significant increase but there was no effect at the 0.3 and 3.0 mg/kg doses compared to vehicle.

At first glance, the dose‐dependent global brain inactivation (negative BOLD VOA) produced by NLX‐112 appears straightforward and consistent with its reported selective 5‐HT_1A_R agonist pharmacology [26]. For example, activation of neuronal 5‐HT_1A_ heteroreceptors that are canonically coupled to a Gα_i/o_ protein generally produces a local inhibition of neuronal activity. However, 5‐HT_1A_ heteroreceptors are expressed on inhibitory (GABAergic) as well as excitatory (glutamatergic) neurons, pre‐ and post‐synaptically [49], complicating the net effect regarding BOLD VOA in a particular brain region [50, 51]. Moreover, although NLX‐112 has no reported physiologically relevant affinity for 5‐HT_2_‐type, adrenergic, and dopamine receptors [12], we could not find any published reports of extensive off‐target profiling for NLX‐112 and its metabolites; thus, region‐specific, as well as global effects, on BOLD VOA could reflect polypharmacology. Certainly, polypharmacology is involved in the global brain activating effects of FPT observed in these studies, as it is known that in addition to 5‐HT_1A_R agonism, FPT is a potent, full‐efficacy agonist at the 5‐HT_1B_ and 5‐HT_1D_Rs, and a potent, partial agonist at the 5‐HT_7_ and adrenergic α_2A_Rs (inverse agonist at α_2C_R), all expressed broadly across the brain.

Despite differences in 5‐HT_1_R subtype pharmacology, as well as other receptor pharmacology differences (known and unknown), both NLX‐112 and FPT have about the same potency and full efficacy at the 5‐HT_1A_R, which are richly expressed in the cerebral cortex [52]. Both compounds produced a positive BOLD VOA along with the complete absence of negative BOLD VOA in the prefrontal cortex, suggesting cognitive function would not be negatively impacted, consistent with current hypotheses that 5‐HT_1A_R activation can enhance cognition [53]. Another common area of concern in CNS‐targeting compounds is abuse liability. There was a strong positive BOLD VOA for both NLX‐112 and FPT in the accumbens and ventral pallidum, regions in the reward circuitry where the 5‐HT_1A_R is abundantly expressed [54]. However, NLX‐112 has not exhibited abuse liability in rats and macaques [55], and there have been no reports of abuse liability arising during its clinical trials for l‐DOPA‐induced dyskinesia (LID) in Parkinson's disease [37]. Moreover, the 5‐HT_1A_R partial agonist buspirone, approved for generalized anxiety disorder in 1986 [10], and its close congener gepirone, approved for major depressive disorder in 2023 [11] have not been associated with abuse liability [56, 57].

The initial studies published regarding NLX‐112 were focused on its potential as a non‐opioid alternative for acute, tonic, and neuropathic pain, and demonstrated robust antinociceptive and analgesic activity in animals [5, 12]. Here, NLX‐112 demonstrated a significant increase in negative BOLD VOA in many brain regions associated with pain neurocircuitry, including the secondary somatosensory cortices, thalamus, hypothalamus, median raphe, spinal trigeminal nucleus, pontine nuclei, and cerebellum. Moreover, the 5‐HT_1A_R is highly expressed in the dorsal horn of the spinal cord [58] and NLX‐112 was studied in a phase 2 clinical trial for diabetic neuropathy, but did not show efficacy, despite preclinical studies that suggested analgesic and antinociceptive properties [5, 12]. Meanwhile, the spinal trigeminal nucleus was the only brain region related to pain where FPT (3.0 mg/kg) produced a negative BOLD VOA.

NLX‐112 also significantly increased negative BOLD VOA in the substantia nigra as well as caudate putamen and several thalamic regions (lateral posterior n. thalamus, and posterior n. thalamus)—these are areas rich in 5‐HT_1A_R (as well as 5‐HT_1B_R) expression [54], which may rationalize the efficacy observed for NLX‐112 in a recent clinical trial for LID in Parkinson's disease [37]. Additionally, there is both negative and positive BOLD signal in the ventral pallidum, a region associated with involuntary movement that has been correlated with LID [59]. Given that the brain regions of interest are composed of heterogeneous voxels, a portion of voxels may have significantly more activation than others, drawing blood flow from neighboring voxels and presenting as both positive and negative BOLD VOA in the same region, that is, the so‐called hemodynamic effect [60, 61]. NLX‐112 also increased positive BOLD VOA in the anterior olfactory n. and piriform ctx, indicating activation of the olfactory system, which expresses the 5‐HT_1A_R [62]. Atrophy of the olfactory system leading to a reduction or loss of smell is one of the first symptoms experienced by 95% of Parkinson's patients [63], and results here suggest NLX‐112 may impact this symptom. In contrast, positive BOLD VOA in the olfactory system was absent with FPT treatment, perhaps reflecting FPT activity at adrenergic α_2C_R which are abundant in this region [64, 65].

FPT showed significant positive BOLD VOA in the prelimbic cortex, medial mammillary nucleus (hypothalamus), and stria terminals, which are connected to emotional and fear regulation [66, 67]. Additionally, there is positive BOLD VOA in the anterior cingulate ctx, a region involved in higher‐level functions, such as attention allocation, reward anticipation, decision making, impulse control, and emotion [68]. The MRI results here are consistent with FPT activation of 5‐HT_1A_ and 5‐HT_1B_Rs in these regions [4, 16, 69], and correlate with FPT's anxiolytic and prosocial effects observed in the autism model utilizing *Fmr1* KO mice [29]. Interestingly, abnormalities in the cerebellum have been linked to autism [70] and FPT showed only positive BOLD signal in cerebellar regions (which express 5‐HT_1_ as well as adrenergic α_2A_Rs [71]), whereas, NLX‐112 demonstrated primarily negative BOLD signal. High levels of α_2a_R (and α_2c_R) also are expressed in the ascending reticular activating system and thalamus, where FPT showed positive BOLD VOA (i.e., lateral paragigantocellular n., medullar reticular dorsal n., and principal sensory n., anterior thalamic n., anterior pretectal thalamic n., and lateral dorsal thalamic n.). These regions are associated with regulating attention, behavior, sensory, and motor signals, thought to be disrupted in autism and ADHD, perhaps due to noradrenergic dysfunction [28]. FPT also showed a positive BOLD signal in other brain regions such as lateral lemniscus and inferior colliculus that may be associated with audiogenic seizures in *Fmr1* KO mice, which are abolished by FPT [29]. Analogously, the lack of a positive BOLD signal for FPT in the gray matter of the hippocampus (CA1 and CA3) is consistent with the anti‐seizure activity of FPT in the *Fmr1* KO mouse model of autism [29, 36, 72, 73, 74] albeit there is positive BOLD signal in the white matter fimbria region of the hippocampus. NLX‐112 increased negative BOLD VOA in the hippocampus, which, like FPT is consistent with its prevention of seizures in *Fmr1* KO mice [36].

It is important to also consider the potential peripheral effects of 5‐HT_1_R agonists when interpreting these phMRI results. Alterations in peripheral vascular tone and blood flow can impact systemic hemodynamics, including cerebral blood flow, which in turn can impact BOLD signal measurements. For example, activation of the 5‐HT_1B_ and 5‐HT_1D_Rs can cause vasoconstriction [75, 76], which can produce a large increase in negative BOLD VOA [77, 78] due to decreased blood flow to the brain with resulting decreased metabolic activity. Although FPT activates 5‐HT_1B_ and 5‐HT_1D_Rs receptors, the overall lack of negative BOLD signal for the FPT treatments suggests there was not a large impact on cerebral blood flow to specific brain regions.

In summary, the acute effects of FPT and NLX‐112 on BOLD VOA were studied in awake mice. Overall, FPT increased global brain activity; however, dose‐related effects were complex, likely reflecting FPT's polypharmacology involving 5‐HT_1_ subtypes and other receptors. In contrast, NLX‐112, which is reported to be a selective 5‐HT_1A_R agonist, broadly inhibited brain activity in a dose‐dependent manner. Neither FPT nor NLX‐112 produced activation of the hippocampus, consistent with their anti‐seizure effects in the *Fmr1* KO mouse model of autism.

### 4.1 Limitations of This Study

We note that phMRI does not provide specific information about drug interaction with known receptor targets. Rather, observed patterns of phMRI BOLD signaling reflect a composite of direct and indirect drug effects on neuronal populations and circuits in the CNS. We discussed compound effects on BOLD VOA here relative to results obtained with the compounds in vivo—notably, we did not include females in the current studies given that male *Fmr1* KO mice (used for both FPT and NLX‐112 in vivo studies) have a heightened susceptibility to audiogenic seizures, resulting in a higher incidence of deaths compared to their female counterparts [79]. Moreover, utilizing both sexes in phMRI studies can add confounding factors due to differences in the sizes of various brain regions which could impact BOLD signal spatial distribution and magnitude. Also, there were technical limitations to including female mice given our analyses required subjects to be registered to the same mouse atlas that does not account for sex‐specific size differences in brain regions.

## Author Contributions

**Brittany M. Brems:** conceptualization, methodology, data curation, investigation, formal analysis, writing – original draft, writing – review and editing. **Erin E. Sullivan:** conceptualization, investigation, writing – original draft, methodology, writing – review and editing, formal analysis, data curation. **Ryan P. McGlynn:** writing – original draft, conceptualization, methodology, investigation, formal analysis. **Praveen Kulkarni:** conceptualization, methodology, software, formal analysis, resources, supervision. **Craig F. Ferris:** writing – review and editing, writing – original draft, conceptualization, methodology, formal analysis, resources, data curation, visualization, supervision, funding acquisition. **Raymond G. Booth:** writing – review and editing, conceptualization, resources, supervision, funding acquisition.

## Funding

This work was supported by the U.S. Department of Defense (Grants W81XWH‐15‐0247 and W81XWH‐17‐1‐0322) and the National Institute on Drug Abuse (Grants R01DA047130 and T32DA055553).

## Ethics Statement

The protocol (#23‐0406R) used in this study complied with the regulations of the Institutional Animal Care and Use Committee at Northeastern University.

## Conflicts of Interest

Craig F. Ferris has a financial interest in Ekam Imaging, a company that makes radiofrequency electronics and holders for awake animal imaging. Craig F. Ferris and Praveen Kulkarni have a partnership interest in Ekam Solutions, a company that develops 3D MRI atlases for animal research. The remaining authors declare that the research was conducted in the absence of any commercial or financial relationships that could be construed as a potential conflict of interest.

## Supporting information


**Data S1:** prp270199‐sup‐0001‐DataS1.docx.

## Data Availability

The original contributions presented in this study are included in the article/Supporting Information; further inquiries can be directed to the corresponding author.
