# Supplementary material for: Pharmacologic MRI Brain Imaging Studies of Serotonin 5‐HT1 Receptor Agonists in Awake Mice
Source: Pharmacol Res Perspect. 2026 Apr 23;14(3):e70199. doi: 10.1002/prp2.70199 (PMC13106894; doi:10.1002/prp2.70199)
Supplement: Supplementary file 1 — Data S1: prp270199‐sup‐0001‐DataS1.docx. [file PRP2-14-e70199-s001.docx]

**Supplemental Material**

**SUPPLEMENTAL TABLE 1a** Positive Volume of Activation of FPT at all doses.

| **FPT Positive VOA** | | | | | | | | | | |
| --- | --- | --- | --- | --- | --- | --- | --- | --- | --- | --- |
|  | **Vehicle** | | **0.03 mg/kg** | | **0.3 mg/kg** | | **3.0 mg/kg** | |  | |
| **Brain Area** | **Mean** | **SE** | **Mean** | **SE** | **Mean** | **SE** | **Mean** | **SE** | **P va** | **Ω Sq** |
| **3rd cerebellar lobule** | **0** | **0.0** | **0** | **0.0** | **14** | **6.6** | **42** | **7.6** | **0.000** | **0.781** |
| **medullary reticular dorsal area** | **56** | **18.7** | **0** | **0.0** | **2** | **1.0** | **7** | **2.1** | **0.000** | **0.725** |
| **5th cerebellar lobule** | **1** | **0.4** | **0** | **0.0** | **45** | **17.4** | **81** | **15.0** | **0.000** | **0.690** |
| **6th cerebellar lobule** | **3** | **1.4** | **0** | **0.0** | **104** | **31.7** | **121** | **24.8** | **0.000** | **0.687** |
| **simple lobule cerebellum** | **24** | **11.3** | **0** | **0.0** | **47** | **13.4** | **91** | **10.6** | **0.000** | **0.667** |
| **crus of ansiform lobule** | **118** | **33.2** | **0** | **0.0** | **128** | **30.0** | **228** | **32.0** | **0.000** | **0.634** |
| **lateral paragigantocellular area** | **17** | **4.6** | **0** | **0.0** | **22** | **3.8** | **39** | **6.0** | **0.000** | **0.606** |
| **paramedian lobule** | **13** | **2.9** | **0** | **0.0** | **29** | **9.7** | **58** | **10.8** | **0.000** | **0.604** |
| **flocculus cerebellum** | **37** | **12.0** | **0** | **0.0** | **61** | **10.8** | **80** | **15.7** | **0.000** | **0.548** |
| **7th cerebellar lobule** | **0** | **0.1** | **0** | **0.0** | **22** | **9.5** | **27** | **8.1** | **0.000** | **0.533** |
| **ambiguus area** | **2** | **1.1** | **0** | **0.0** | **3** | **1.1** | **7** | **1.5** | **0.000** | **0.528** |
| **vestibular area** | **10** | **4.6** | **0** | **0.0** | **36** | **13.8** | **55** | **14.8** | **0.000** | **0.523** |
| **pontine reticular nucleus caudal** | **18** | **8.5** | **0** | **0.0** | **49** | **11.8** | **51** | **21.8** | **0.000** | **0.517** |
| **8th cerebellar lobule** | **54** | **11.9** | **0** | **0.0** | **12** | **5.0** | **16** | **10.5** | **0.000** | **0.505** |
| **spinal trigeminal nuclear area** | **36** | **15.0** | **0** | **0.0** | **55** | **13.2** | **63** | **14.5** | **0.000** | **0.497** |
| **intermediate reticular area** | **16** | **4.9** | **0** | **0.0** | **22** | **7.1** | **34** | **8.7** | **0.000** | **0.489** |
| **medullary reticular ventral area** | **56** | **19.1** | **0** | **0.0** | **8** | **4.1** | **16** | **5.5** | **0.000** | **0.487** |
| **stria terminalis** | **18** | **6.0** | **0** | **0.0** | **0** | **0.1** | **0** | **0.1** | **0.000** | **0.478** |
| **gigantocelllaris reticular area** | **20** | **6.7** | **0** | **0.0** | **72** | **22.9** | **84** | **35.0** | **0.000** | **0.472** |
| **fimbria hippocampus** | **13** | **3.0** | **11** | **2.8** | **29** | **7.3** | **41** | **4.6** | **0.001** | **0.458** |
| **2nd cerebellar lobule** | **13** | **3.3** | **0** | **0.0** | **11** | **4.0** | **34** | **11.9** | **0.001** | **0.456** |
| **pyramidal tracts** | **5** | **2.0** | **3** | **1.4** | **27** | **5.4** | **18** | **5.9** | **0.001** | **0.439** |
| **pedunculopontine tegmental area** | **12** | **5.1** | **0** | **0.0** | **15** | **5.0** | **32** | **8.5** | **0.001** | **0.432** |
| **principal sensory nucleus trigeminal** | **13** | **6.3** | **16** | **4.9** | **48** | **9.0** | **55** | **11.9** | **0.001** | **0.426** |
| **olivary complex** | **7** | **3.4** | **0** | **0.0** | **18** | **2.8** | **11** | **4.5** | **0.002** | **0.385** |
| **solitary tract area** | **19** | **4.9** | **0** | **0.0** | **24** | **8.1** | **21** | **8.6** | **0.002** | **0.374** |
| **locus ceruleus** | **0** | **0.0** | **0** | **0.0** | **1** | **0.4** | **1** | **0.5** | **0.003** | **0.358** |
| **cerebellar nuclear area** | **2** | **0.5** | **0** | **0.0** | **11** | **4.2** | **22** | **10.3** | **0.003** | **0.356** |
| **parvicellular reticular area** | **15** | **4.8** | **0** | **0.0** | **27** | **8.0** | **24** | **9.4** | **0.003** | **0.356** |
| **optic tract** | **2** | **0.8** | **1** | **0.4** | **7** | **1.1** | **3** | **1.2** | **0.003** | **0.349** |
| **prelimbic ctx** | **2** | **1.5** | **9** | **4.9** | **4** | **3.4** | **24** | **8.2** | **0.003** | **0.343** |
| **accumbens shell** | **1** | **0.6** | **18** | **6.6** | **19** | **4.2** | **21** | **4.8** | **0.003** | **0.338** |
| **zona incerta** | **10** | **2.8** | **4** | **1.4** | **19** | **3.8** | **15** | **2.2** | **0.004** | **0.050** |
| **accumbens core** | **1** | **0.9** | **8** | **4.6** | **3** | **2.4** | **11** | **4.0** | **0.005** | **0.306** |
| **entorhinal ctx** | **199** | **32.3** | **148** | **14.7** | **247** | **25.9** | **272** | **27.3** | **0.006** | **0.299** |
| **orbital ctx** | **11** | **3.9** | **25** | **8.5** | **21** | **9.7** | **74** | **24.6** | **0.007** | **0.284** |
| **facial nucleus** | **9** | **3.5** | **0** | **0.0** | **5** | **1.2** | **5** | **1.9** | **0.007** | **0.284** |
| **frontal association ctx** | **2** | **0.6** | **7** | **3.0** | **5** | **3.2** | **32** | **13.7** | **0.008** | **0.282** |
| **tenia tecta ctx** | **4** | **2.1** | **24** | **6.3** | **20** | **4.7** | **28** | **3.0** | **0.008** | **0.279** |
| **secondary motor ctx** | **31** | **11.0** | **25** | **9.3** | **65** | **19.2** | **78** | **12.9** | **0.008** | **0.275** |
| **inferior colliculus** | **89** | **26.0** | **52** | **8.7** | **150** | **33.7** | **179** | **27.3** | **0.009** | **0.035** |
| **lateral reticular area** | **2** | **2.3** | **0** | **0.0** | **5** | **2.8** | **8** | **2.9** | **0.009** | **0.268** |
| **10th cerebellar lobule** | **0** | **0.1** | **0** | **0.0** | **6** | **3.2** | **13** | **5.7** | **0.010** | **0.265** |
| **9th cerebellar lobule** | **17** | **4.6** | **0** | **0.0** | **12** | **5.7** | **11** | **7.0** | **0.010** | **0.263** |
| **prepositus area** | **0** | **0.1** | **0** | **0.0** | **4** | **1.9** | **6** | **2.3** | **0.010** | **0.261** |
| **lateral lemniscus** | **4** | **2.6** | **3** | **2.2** | **10** | **2.3** | **12** | **2.8** | **0.012** | **0.250** |
| **lateral rostral hypothalamic area** | **22** | **4.1** | **29** | **8.3** | **68** | **11.9** | **51** | **10.4** | **0.012** | **0.249** |
| **parabrachial area** | **5** | **2.2** | **0** | **0.0** | **7** | **2.5** | **9** | **3.5** | **0.013** | **0.243** |
| **4th cerebellar lobule** | **9** | **3.6** | **0** | **0.0** | **11** | **4.3** | **18** | **6.5** | **0.014** | **0.239** |
| **dorsal hippocampal commissure** | **4** | **1.6** | **2** | **0.8** | **5** | **2.1** | **9** | **1.5** | **0.016** | **0.233** |
| **substantia nigra** | **16** | **5.2** | **5** | **1.3** | **19** | **7.8** | **32** | **7.4** | **0.016** | **0.229** |
| **granular cell layer** | **5** | **1.5** | **22** | **12.9** | **15** | **6.0** | **34** | **6.9** | **0.018** | **0.223** |
| **ventral pallidum** | **5** | **2.5** | **19** | **7.6** | **31** | **7.3** | **23** | **7.0** | **0.018** | **0.222** |
| **primary motor ctx** | **30** | **11.5** | **18** | **6.3** | **52** | **16.0** | **59** | **8.7** | **0.019** | **0.217** |
| **anterior thalamic area** | **5** | **2.0** | **8** | **3.7** | **9** | **4.1** | **19** | **4.4** | **0.020** | **0.215** |
| **retrosplenial rostral ctx** | **42** | **14.5** | **33** | **14.1** | **83** | **13.4** | **79** | **10.1** | **0.020** | **0.037** |
| **medial preoptic area** | **9** | **3.1** | **17** | **6.9** | **26** | **4.1** | **27** | **6.2** | **0.023** | **0.207** |
| **anterior cingulate area** | **15** | **4.3** | **8** | **4.0** | **25** | **9.5** | **37** | **8.2** | **0.023** | **0.205** |
| **anterior pretectal thalamic area** | **5** | **1.7** | **5** | **1.0** | **9** | **3.0** | **12** | **2.3** | **0.025** | **0.201** |
| **basal amygdaloid area** | **29** | **5.5** | **31** | **10.3** | **63** | **5.2** | **50** | **13.4** | **0.026** | **0.196** |
| **anterior commissure** | **0** | **0.3** | **3** | **1.9** | **1** | **1.0** | **3** | **1.7** | **0.029** | **0.190** |
| **medial mammillary area** | **6** | **2.8** | **12** | **3.5** | **9** | **5.0** | **19** | **3.3** | **0.032** | **0.184** |
| **lateral caudal hypothalamic area** | **16** | **4.4** | **11** | **2.0** | **24** | **7.3** | **27** | **5.6** | **0.034** | **0.179** |
| **retrosplenial caudal ctx** | **69** | **17.2** | **47** | **12.6** | **99** | **19.3** | **93** | **12.5** | **0.041** | **0.014** |
| **cuneate area** | **3** | **2.2** | **0** | **0.0** | **1** | **0.6** | **2** | **0.9** | **0.048** | **0.155** |
| **CA1** | **81** | **28.1** | **49** | **15.3** | **138** | **27.9** | **132** | **29.4** | **0.053** | **0.148** |
| **lateral dorsal thalamic area** | **5** | **1.5** | **5** | **1.8** | **12** | **4.4** | **15** | **3.8** | **0.055** | **0.011** |
| **extended amydala** | **4** | **2.0** | **2** | **1.0** | **11** | **2.8** | **5** | **2.8** | **0.056** | **0.144** |
| **cerebral peduncle** | **31** | **12.1** | **37** | **15.8** | **63** | **16.7** | **88** | **25.0** | **0.059** | **0.140** |
| **lateral amygdaloid area** | **4** | **1.8** | **3** | **1.5** | **8** | **1.9** | **4** | **1.3** | **0.064** | **0.134** |
| **diagonal band of Broca** | **3** | **1.1** | **10** | **4.0** | **12** | **3.3** | **12** | **3.3** | **0.068** | **0.129** |
| **medial geniculate** | **20** | **5.8** | **13** | **3.7** | **29** | **5.5** | **27** | **4.9** | **0.078** | **0.120** |
| **endopiriform area** | **0** | **0.2** | **8** | **3.3** | **3** | **2.3** | **4** | **1.6** | **0.089** | **0.110** |
| **lemniscal area** | **6** | **3.4** | **6** | **3.1** | **8** | **3.3** | **19** | **5.4** | **0.091** | **0.109** |
| **anterior amygdaloid area** | **2** | **0.7** | **7** | **3.6** | **5** | **1.5** | **10** | **2.4** | **0.094** | **0.107** |
| **caudal piriform ctx** | **76** | **10.3** | **63** | **11.0** | **98** | **13.2** | **61** | **9.2** | **0.094** | **0.107** |
| **glomerular layer** | **23** | **4.9** | **26** | **12.6** | **53** | **10.5** | **32** | **10.5** | **0.097** | **0.104** |
| **dorsal medial hypothalamic area** | **2** | **1.1** | **4** | **1.5** | **7** | **1.7** | **7** | **1.9** | **0.113** | **0.093** |
| **rostral piriform ctx** | **20** | **7.4** | **79** | **37.9** | **66** | **18.9** | **34** | **7.0** | **0.113** | **0.093** |
| **subiculum** | **87** | **24.6** | **49** | **10.2** | **133** | **28.4** | **129** | **34.8** | **0.114** | **0.093** |
| **ventral medial hypothalamic area** | **9** | **3.9** | **21** | **5.0** | **7** | **3.9** | **15** | **4.1** | **0.115** | **0.092** |
| **pituitary** | **4** | **2.0** | **18** | **5.7** | **14** | **4.8** | **14** | **4.4** | **0.119** | **0.090** |
| **corpus callosum** | **38** | **7.9** | **29** | **7.7** | **63** | **14.7** | **60** | **11.2** | **0.121** | **0.088** |
| **lateral geniculate** | **12** | **4.5** | **3** | **1.5** | **14** | **3.2** | **8** | **1.7** | **0.121** | **0.045** |
| **pontine area** | **12** | **8.7** | **26** | **9.9** | **39** | **11.4** | **27** | **14.4** | **0.125** | **0.086** |
| **insular caudal ctx** | **28** | **8.1** | **19** | **7.0** | **33** | **6.5** | **16** | **3.9** | **0.128** | **0.084** |
| **visual 1 ctx** | **199** | **32.2** | **190** | **17.8** | **186** | **18.5** | **143** | **20.5** | **0.146** | **0.075** |
| **interpeduncular area** | **2** | **1.3** | **1** | **1.0** | **2** | **1.1** | **7** | **3.1** | **0.166** | **0.065** |
| **parietal ctx** | **2** | **1.4** | **3** | **1.8** | **5** | **1.3** | **4** | **1.8** | **0.189** | **0.000** |
| **ventral tegmental area** | **3** | **1.4** | **2** | **0.9** | **4** | **2.4** | **9** | **3.5** | **0.192** | **0.054** |
| **ventricle** | **38** | **12.5** | **37** | **6.3** | **77** | **23.1** | **75** | **18.4** | **0.193** | **0.054** |
| **ventral thalamic area** | **36** | **18.4** | **28** | **12.8** | **58** | **16.6** | **45** | **10.3** | **0.212** | **0.012** |
| **dorsal raphe** | **6** | **3.1** | **3** | **1.4** | **9** | **2.6** | **12** | **4.1** | **0.214** | **0.043** |
| **reticular thalamic area** | **9** | **4.3** | **4** | **1.1** | **10** | **3.1** | **10** | **2.2** | **0.234** | **0.040** |
| **temporal ctx** | **11** | **3.1** | **18** | **3.7** | **19** | **6.0** | **26** | **6.7** | **0.244** | **0.037** |
| **periaqueductal gray** | **56** | **18.8** | **34** | **8.7** | **83** | **23.0** | **93** | **29.8** | **0.247** | **0.036** |
| **mesencephalic reticular formation** | **71** | **23.9** | **39** | **11.0** | **78** | **22.2** | **109** | **32.8** | **0.248** | **0.035** |
| **lateral preoptic area** | **2** | **0.9** | **4** | **1.7** | **6** | **2.1** | **3** | **1.7** | **0.261** | **0.032** |
| **superior colliculus** | **128** | **40.5** | **78** | **21.1** | **188** | **44.4** | **168** | **43.5** | **0.263** | **0.047** |
| **forceps minor corpus callosum** | **2** | **0.6** | **2** | **1.2** | **2** | **0.6** | **3** | **1.4** | **0.298** | **0.021** |
| **lateral posterior thalamic area** | **8** | **3.8** | **8** | **2.9** | **16** | **4.7** | **14** | **4.7** | **0.304** | **0.056** |
| **medial amygdaloid area** | **16** | **4.5** | **15** | **5.7** | **28** | **6.0** | **30** | **10.6** | **0.318** | **0.016** |
| **primary somatosensory ctx** | **172** | **40.1** | **116** | **21.7** | **253** | **59.3** | **202** | **48.6** | **0.327** | **0.014** |
| **caudate putamen** | **97** | **20.6** | **91** | **28.4** | **145** | **44.9** | **141** | **21.1** | **0.357** | **0.007** |
| **anterior olfactory area** | **24** | **5.3** | **57** | **15.7** | **40** | **9.0** | **38** | **10.3** | **0.359** | **0.007** |
| **posterior thalamic area** | **8** | **3.7** | **7** | **3.4** | **8** | **3.0** | **14** | **3.6** | **0.374** | **0.015** |
| **external capsule** | **10** | **2.6** | **8** | **3.3** | **16** | **4.8** | **16** | **4.7** | **0.388** | **0.001** |
| **decussation superior cerebellar peduncle** | **1** | **0.5** | **0** | **0.3** | **0** | **0.0** | **1** | **0.4** | **0.397** | **0.151** |
| **CA3** | **34** | **11.4** | **19** | **4.8** | **46** | **12.6** | **45** | **13.6** | **0.400** | **0.013** |
| **olfactory tubercles** | **18** | **6.4** | **32** | **7.6** | **30** | **6.2** | **26** | **4.7** | **0.405** | **0.003** |
| **globus pallidus** | **16** | **6.7** | **11** | **5.3** | **16** | **6.0** | **19** | **5.5** | **0.421** | **0.006** |
| **reuniens thalamic area** | **4** | **1.9** | **5** | **3.4** | **8** | **3.0** | **8** | **3.4** | **0.453** | **0.054** |
| **secondary somaotsensory ctx** | **54** | **16.0** | **28** | **11.8** | **62** | **23.0** | **48** | **10.9** | **0.480** | **0.016** |
| **habenular area** | **3** | **0.9** | **5** | **1.1** | **3** | **1.4** | **2** | **0.9** | **0.491** | **0.018** |
| **internal capsule** | **17** | **5.1** | **11** | **3.3** | **17** | **4.4** | **19** | **2.5** | **0.496** | **0.025** |
| **anterior hypothalamic area** | **28** | **7.8** | **42** | **7.8** | **40** | **9.7** | **44** | **6.4** | **0.497** | **0.019** |
| **lateral septal area** | **12** | **2.7** | **19** | **8.3** | **28** | **11.0** | **25** | **6.9** | **0.550** | **0.028** |
| **cortical amygdaloid area** | **53** | **7.7** | **36** | **9.9** | **45** | **4.3** | **45** | **6.2** | **0.567** | **0.031** |
| **dentate gyrus** | **87** | **28.6** | **60** | **18.6** | **118** | **26.7** | **112** | **33.3** | **0.579** | **0.014** |
| **central amygdaloid area** | **13** | **5.5** | **10** | **4.8** | **21** | **7.5** | **16** | **7.1** | **0.594** | **0.034** |
| **pontine reticular nucleus oral** | **30** | **12.6** | **25** | **9.9** | **31** | **8.5** | **48** | **18.0** | **0.595** | **0.035** |
| **median raphe area** | **14** | **6.1** | **7** | **4.3** | **15** | **7.5** | **16** | **8.1** | **0.599** | **0.035** |
| **paraventricular thalamic area** | **2** | **1.1** | **5** | **3.0** | **4** | **2.4** | **6** | **2.5** | **0.684** | **0.028** |
| **fornix** | **0** | **0.1** | **1** | **0.3** | **0** | **0.1** | **1** | **0.5** | **0.698** | **0.049** |
| **bed nucleus stria terminalis** | **3** | **1.5** | **10** | **4.7** | **9** | **7.3** | **7** | **3.3** | **0.733** | **0.054** |
| **infralimbic ctx** | **1** | **0.3** | **2** | **1.1** | **1** | **0.3** | **3** | **1.4** | **0.761** | **0.057** |
| **reticulotegmental nucleus** | **3** | **2.2** | **3** | **1.5** | **2** | **1.0** | **8** | **6.0** | **0.786** | **0.061** |
| **medial dorsal thalamic area** | **5** | **1.6** | **8** | **1.9** | **7** | **3.0** | **10** | **4.2** | **0.787** | **0.061** |
| **medial septal area** | **1** | **0.3** | **2** | **1.4** | **1** | **1.0** | **2** | **1.5** | **0.801** | **0.063** |
| **auditory ctx** | **35** | **6.3** | **35** | **8.9** | **41** | **9.4** | **53** | **14.7** | **0.819** | **0.065** |
| **posterior hypothalamic area** | **3** | **1.4** | **3** | **1.1** | **6** | **2.3** | **5** | **2.1** | **0.830** | **0.066** |
| **paraventricular hypothalamic area** | **2** | **1.0** | **1** | **0.8** | **2** | **0.9** | **2** | **1.1** | **0.851** | **0.018** |
| **insular rostral ctx** | **25** | **7.7** | **46** | **16.5** | **40** | **18.4** | **21** | **5.6** | **0.886** | **0.074** |
| **parafascicular thalamic area** | **1** | **0.4** | **1** | **0.6** | **1** | **0.3** | **2** | **1.2** | **0.997** | **0.092** |
| **central medial thalamic area** | **5** | **2.3** | **5** | **2.8** | **4** | **2.0** | **5** | **2.8** | **0.998** | **0.093** |

**SUPPLEMENTAL TABLE 1b** Negative Volume of Activation of FPT at all doses.

| **FPT Negative VOA** | | | | | | | | | | |
| --- | --- | --- | --- | --- | --- | --- | --- | --- | --- | --- |
|  | **Vehicle** | | **0.03 mg/kg** | | **0.3 mg/kg** | | **3.0 mg/kg** | |  | |
| **Brain Area** | **Mean** | **SE** | **Mean** | **SE** | **Mean** | **SE** | **Mean** | **SE** | **P va** | **Ω Sq** |
| **ambiguus area** | **70** | **23.6** | **0** | **0.0** | **3** | **1.0** | **6** | **1.7** | **0.000** | **0.646** |
| **medullary reticular dorsal area** | **38** | **11.8** | **0** | **0.0** | **4** | **2.4** | **6** | **2.5** | **0.000** | **0.647** |
| **crus of ansiform lobule** | **77** | **18.7** | **0** | **0.0** | **151** | **27.5** | **86** | **16.8** | **0.000** | **0.560** |
| **lateral reticular area** | **31** | **7.4** | **0** | **0.0** | **2** | **1.3** | **10** | **3.4** | **0.000** | **0.518** |
| **lateral rostral hypothalamic area** | **14** | **4.7** | **7** | **2.5** | **25** | **5.8** | **2** | **0.8** | **0.000** | **0.481** |
| **paramedian lobule** | **10** | **4.1** | **0** | **0.0** | **37** | **10.5** | **22** | **5.6** | **0.000** | **0.517** |
| **spinal trigeminal nuclear area** | **12** | **4.6** | **0** | **0.0** | **34** | **10.1** | **61** | **17.3** | **0.000** | **0.496** |
| **simple lobule cerebellum** | **26** | **7.9** | **0** | **0.0** | **45** | **13.2** | **21** | **4.0** | **0.000** | **0.509** |
| **gigantocelllaris reticular area** | **16** | **4.9** | **0** | **0.0** | **89** | **25.4** | **49** | **17.5** | **0.000** | **0.485** |
| **flocculus cerebellum** | **34** | **11.7** | **0** | **0.0** | **50** | **9.8** | **39** | **12.5** | **0.000** | **0.481** |
| **intermediate reticular area** | **7** | **3.7** | **0** | **0.0** | **29** | **8.9** | **24** | **9.0** | **0.001** | **0.465** |
| **endopiriform area** | **15** | **3.2** | **1** | **0.3** | **23** | **6.1** | **9** | **1.5** | **0.001** | **0.426** |
| **pontine reticular nucleus caudal** | **35** | **14.8** | **0** | **0.0** | **71** | **20.0** | **29** | **8.9** | **0.001** | **0.452** |
| **lateral paragigantocellular area** | **5** | **1.7** | **0** | **0.0** | **14** | **4.5** | **5** | **1.9** | **0.001** | **0.449** |
| **cerebellar nuclear area** | **3** | **1.1** | **0** | **0.0** | **29** | **12.1** | **8** | **3.5** | **0.001** | **0.421** |
| **rostral piriform ctx** | **93** | **27.7** | **17** | **9.3** | **151** | **24.6** | **71** | **11.2** | **0.001** | **0.394** |
| **vestibular area** | **12** | **7.2** | **0** | **0.0** | **53** | **18.2** | **15** | **4.2** | **0.001** | **0.435** |
| **auditory ctx** | **41** | **7.6** | **17** | **5.6** | **73** | **10.5** | **38** | **7.4** | **0.001** | **0.358** |
| **stria terminalis** | **6** | **4.7** | **0** | **0.0** | **0** | **0.0** | **0** | **0.1** | **0.001** | **0.390** |
| **entorhinal ctx** | **146** | **29.4** | **71** | **20.5** | **220** | **22.8** | **120** | **12.6** | **0.002** | **0.375** |
| **6th cerebellar lobule** | **23** | **10.0** | **0** | **0.0** | **32** | **15.1** | **39** | **10.0** | **0.002** | **0.410** |
| **caudate putamen** | **286** | **38.8** | **116** | **32.1** | **417** | **86.0** | **128** | **41.1** | **0.002** | **0.342** |
| **3rd cerebellar lobule** | **2** | **1.2** | **0** | **0.0** | **23** | **9.6** | **1** | **0.7** | **0.002** | **0.411** |
| **2nd cerebellar lobule** | **2** | **1.1** | **0** | **0.0** | **38** | **13.8** | **12** | **4.5** | **0.002** | **0.374** |
| **glomerular layer** | **162** | **26.4** | **76** | **39.5** | **222** | **38.3** | **66** | **8.6** | **0.002** | **0.341** |
| **caudal piriform ctx** | **48** | **8.2** | **18** | **4.7** | **79** | **15.6** | **37** | **6.9** | **0.002** | **0.340** |
| **medial amygdaloid area** | **28** | **7.5** | **3** | **0.8** | **34** | **7.4** | **15** | **5.5** | **0.003** | **0.322** |
| **parvicellular reticular area** | **14** | **3.8** | **0** | **0.0** | **20** | **6.2** | **21** | **7.8** | **0.003** | **0.349** |
| **pituitary** | **10** | **5.3** | **0** | **0.0** | **18** | **5.3** | **18** | **3.8** | **0.003** | **0.309** |
| **olivary complex** | **4** | **2.0** | **0** | **0.0** | **7** | **2.1** | **10** | **3.2** | **0.004** | **0.327** |
| **solitary tract area** | **23** | **9.0** | **0** | **0.0** | **14** | **6.0** | **20** | **8.0** | **0.005** | **0.318** |
| **pedunculopontine tegmental area** | **12** | **6.0** | **0** | **0.0** | **23** | **8.4** | **10** | **3.5** | **0.006** | **0.306** |
| **bed nucleus stria terminalis** | **16** | **5.2** | **14** | **5.1** | **37** | **11.3** | **2** | **1.6** | **0.007** | **0.264** |
| **basal amygdaloid area** | **40** | **6.6** | **10** | **3.7** | **43** | **7.2** | **37** | **9.0** | **0.007** | **0.281** |
| **medullary reticular ventral area** | **37** | **18.0** | **0** | **0.0** | **7** | **5.1** | **18** | **8.6** | **0.007** | **0.307** |
| **granular cell layer** | **113** | **21.0** | **50** | **29.4** | **176** | **38.4** | **55** | **9.3** | **0.008** | **0.276** |
| **8th cerebellar lobule** | **56** | **19.5** | **0** | **0.0** | **18** | **10.8** | **18** | **8.9** | **0.008** | **0.299** |
| **7th cerebellar lobule** | **2** | **0.9** | **0** | **0.0** | **15** | **7.2** | **16** | **8.4** | **0.008** | **0.305** |
| **9th cerebellar lobule** | **5** | **1.7** | **0** | **0.0** | **12** | **8.1** | **16** | **6.4** | **0.009** | **0.275** |
| **visual 1 ctx** | **141** | **33.4** | **74** | **15.3** | **199** | **31.6** | **174** | **24.0** | **0.009** | **0.250** |
| **substantia nigra** | **5** | **2.8** | **2** | **2.4** | **15** | **6.4** | **0** | **0.2** | **0.009** | **0.231** |
| **lateral septal area** | **48** | **8.0** | **22** | **9.3** | **40** | **12.3** | **12** | **7.8** | **0.010** | **0.236** |
| **10th cerebellar lobule** | **2** | **0.9** | **0** | **0.0** | **16** | **6.6** | **2** | **0.6** | **0.010** | **0.271** |
| **facial nucleus** | **20** | **7.2** | **0** | **0.0** | **3** | **0.9** | **4** | **1.6** | **0.010** | **0.258** |
| **prepositus area** | **2** | **0.9** | **0** | **0.0** | **7** | **3.2** | **7** | **2.3** | **0.012** | **0.262** |
| **medial mammillary area** | **9** | **4.5** | **0** | **0.1** | **20** | **6.3** | **3** | **1.3** | **0.013** | **0.233** |
| **insular caudal ctx** | **24** | **8.3** | **14** | **3.5** | **36** | **6.8** | **8** | **2.3** | **0.013** | **0.230** |
| **pontine area** | **28** | **12.6** | **1** | **1.1** | **32** | **9.5** | **40** | **11.0** | **0.015** | **0.221** |
| **orbital ctx** | **104** | **22.4** | **47** | **15.9** | **148** | **33.3** | **47** | **17.0** | **0.016** | **0.236** |
| **pontine reticular nucleus oral** | **42** | **19.4** | **3** | **1.2** | **58** | **20.7** | **7** | **2.4** | **0.018** | **0.198** |
| **central amygdaloid area** | **29** | **8.5** | **4** | **2.0** | **26** | **6.2** | **15** | **6.9** | **0.018** | **0.202** |
| **accumbens shell** | **15** | **3.6** | **2** | **0.7** | **15** | **4.9** | **8** | **1.7** | **0.020** | **0.228** |
| **cortical amygdaloid area** | **42** | **10.5** | **28** | **9.1** | **77** | **11.7** | **33** | **6.3** | **0.020** | **0.193** |
| **anterior olfactory area** | **102** | **16.0** | **52** | **22.8** | **188** | **44.1** | **60** | **11.0** | **0.023** | **0.187** |
| **tenia tecta ctx** | **18** | **4.7** | **6** | **3.1** | **23** | **5.2** | **8** | **3.3** | **0.026** | **0.205** |
| **ventral medial hypothalamic area** | **10** | **3.6** | **1** | **0.8** | **16** | **4.8** | **14** | **3.5** | **0.027** | **0.185** |
| **lemniscal area** | **7** | **4.0** | **1** | **1.1** | **11** | **4.4** | **1** | **0.8** | **0.027** | **0.174** |
| **ventricle** | **95** | **19.2** | **41** | **7.0** | **96** | **29.1** | **32** | **8.7** | **0.027** | **0.181** |
| **corpus callosum** | **49** | **11.3** | **49** | **11.2** | **64** | **16.4** | **16** | **6.1** | **0.028** | **0.183** |
| **olfactory tubercles** | **14** | **4.9** | **7** | **2.9** | **23** | **4.1** | **20** | **3.4** | **0.028** | **0.175** |
| **medial septal area** | **4** | **0.9** | **2** | **1.0** | **7** | **2.2** | **2** | **1.3** | **0.029** | **0.182** |
| **accumbens core** | **18** | **4.2** | **3** | **1.8** | **30** | **9.3** | **7** | **2.3** | **0.033** | **0.198** |
| **ventral pallidum** | **14** | **5.1** | **4** | **1.6** | **22** | **6.0** | **16** | **3.5** | **0.035** | **0.182** |
| **cuneate area** | **1** | **0.6** | **0** | **0.0** | **1** | **0.5** | **4** | **1.6** | **0.035** | **0.157** |
| **infralimbic ctx** | **3** | **0.9** | **1** | **0.4** | **2** | **0.8** | **1** | **1.0** | **0.035** | **0.167** |
| **parabrachial area** | **4** | **1.9** | **0** | **0.0** | **9** | **3.5** | **1** | **0.5** | **0.039** | **0.162** |
| **anterior hypothalamic area** | **29** | **8.6** | **12** | **4.8** | **40** | **7.0** | **18** | **5.8** | **0.039** | **0.151** |
| **optic tract** | **2** | **0.9** | **1** | **0.8** | **2** | **1.0** | **0** | **0.1** | **0.039** | **0.162** |
| **prelimbic ctx** | **23** | **6.1** | **10** | **5.2** | **42** | **9.8** | **17** | **6.5** | **0.040** | **0.186** |
| **subiculum** | **38** | **27.0** | **13** | **7.2** | **71** | **22.8** | **21** | **6.8** | **0.040** | **0.160** |
| **reticulotegmental nucleus** | **13** | **5.6** | **1** | **0.8** | **17** | **6.4** | **5** | **2.8** | **0.043** | **0.150** |
| **ventral tegmental area** | **3** | **1.8** | **1** | **1.1** | **7** | **2.8** | **0** | **0.3** | **0.043** | **0.111** |
| **frontal association ctx** | **62** | **10.3** | **32** | **12.5** | **90** | **23.6** | **25** | **6.1** | **0.044** | **0.173** |
| **4th cerebellar lobule** | **6** | **3.4** | **0** | **0.0** | **11** | **4.5** | **3** | **1.6** | **0.045** | **0.155** |
| **anterior amygdaloid area** | **5** | **2.2** | **2** | **1.3** | **10** | **1.8** | **6** | **2.2** | **0.051** | **0.146** |
| **habenular area** | **4** | **0.9** | **1** | **0.4** | **2** | **1.0** | **5** | **1.5** | **0.052** | **0.134** |
| **dorsal hippocampal commissure** | **2** | **1.1** | **2** | **1.2** | **4** | **1.4** | **0** | **0.2** | **0.052** | **0.131** |
| **lateral lemniscus** | **8** | **3.9** | **1** | **0.6** | **10** | **3.7** | **2** | **1.0** | **0.058** | **0.142** |
| **principal sensory nucleus trigeminal** | **29** | **12.2** | **4** | **1.9** | **35** | **12.8** | **13** | **3.9** | **0.061** | **0.163** |
| **dorsal medial hypothalamic area** | **4** | **1.5** | **2** | **1.3** | **1** | **0.6** | **1** | **0.4** | **0.062** | **0.116** |
| **lateral preoptic area** | **2** | **1.4** | **1** | **0.5** | **2** | **1.0** | **0** | **0.0** | **0.065** | **0.088** |
| **interpeduncular area** | **5** | **2.5** | **1** | **0.3** | **10** | **3.9** | **1** | **1.1** | **0.066** | **0.126** |
| **temporal ctx** | **24** | **4.8** | **11** | **4.5** | **28** | **3.7** | **20** | **2.8** | **0.067** | **0.122** |
| **insular rostral ctx** | **70** | **20.3** | **36** | **13.6** | **146** | **35.3** | **38** | **8.3** | **0.080** | **0.101** |
| **globus pallidus** | **21** | **6.0** | **12** | **3.9** | **49** | **12.4** | **19** | **6.4** | **0.085** | **0.103** |
| **lateral caudal hypothalamic area** | **7** | **2.8** | **1** | **0.8** | **8** | **3.1** | **0** | **0.2** | **0.090** | **0.096** |
| **5th cerebellar lobule** | **3** | **1.3** | **0** | **0.0** | **63** | **26.3** | **8** | **5.3** | **0.100** | **0.146** |
| **anterior cingulate area** | **40** | **9.5** | **34** | **11.2** | **59** | **19.3** | **18** | **10.3** | **0.110** | **0.106** |
| **lateral amygdaloid area** | **8** | **3.0** | **2** | **0.9** | **5** | **1.4** | **7** | **2.3** | **0.116** | **0.095** |
| **fornix** | **1** | **0.4** | **0** | **0.3** | **1** | **0.5** | **0** | **0.3** | **0.123** | **0.111** |
| **medial preoptic area** | **17** | **6.2** | **13** | **4.1** | **18** | **3.4** | **7** | **3.1** | **0.144** | **0.087** |
| **posterior hypothalamic area** | **2** | **0.9** | **0** | **0.1** | **2** | **1.2** | **1** | **1.2** | **0.154** | **0.020** |
| **median raphe area** | **16** | **6.9** | **2** | **1.3** | **19** | **7.5** | **4** | **1.5** | **0.156** | **0.056** |
| **fimbria hippocampus** | **25** | **6.2** | **15** | **3.7** | **23** | **8.0** | **9** | **5.0** | **0.171** | **0.098** |
| **central medial thalamic area** | **6** | **2.5** | **1** | **1.3** | **7** | **2.9** | **6** | **2.5** | **0.193** | **0.032** |
| **extended amydala** | **6** | **3.0** | **2** | **0.7** | **12** | **4.0** | **4** | **1.7** | **0.202** | **0.060** |
| **locus ceruleus** | **3** | **2.1** | **0** | **0.0** | **1** | **0.5** | **1** | **0.4** | **0.207** | **0.070** |
| **CA1** | **67** | **29.8** | **26** | **9.8** | **87** | **23.7** | **44** | **14.6** | **0.210** | **0.057** |
| **anterior commissure** | **4** | **1.2** | **4** | **1.5** | **6** | **1.7** | **2** | **1.0** | **0.215** | **0.072** |
| **reticular thalamic area** | **7** | **2.4** | **4** | **2.7** | **12** | **3.6** | **8** | **2.3** | **0.225** | **0.042** |
| **parafascicular thalamic area** | **2** | **0.6** | **1** | **0.5** | **1** | **0.7** | **2** | **0.6** | **0.234** | **0.031** |
| **primary somatosensory ctx** | **185** | **46.4** | **169** | **42.1** | **389** | **84.7** | **186** | **28.6** | **0.240** | **0.036** |
| **secondary somaotsensory ctx** | **46** | **17.4** | **34** | **9.8** | **106** | **27.7** | **45** | **8.6** | **0.252** | **0.030** |
| **medial dorsal thalamic area** | **8** | **2.4** | **3** | **2.2** | **8** | **3.1** | **8** | **3.4** | **0.253** | **0.019** |
| **diagonal band of Broca** | **6** | **2.8** | **4** | **2.2** | **10** | **2.6** | **4** | **1.7** | **0.261** | **0.039** |
| **primary motor ctx** | **68** | **18.5** | **51** | **17.2** | **104** | **29.1** | **41** | **9.7** | **0.273** | **0.045** |
| **secondary motor ctx** | **62** | **19.2** | **52** | **16.7** | **98** | **24.2** | **42** | **9.0** | **0.277** | **0.049** |
| **mesencephalic reticular formation** | **58** | **32.5** | **19** | **9.2** | **75** | **26.1** | **16** | **5.9** | **0.277** | **0.026** |
| **cerebral peduncle** | **32** | **12.5** | **31** | **17.0** | **86** | **30.7** | **26** | **7.4** | **0.277** | **0.037** |
| **periaqueductal gray** | **64** | **24.6** | **19** | **8.7** | **64** | **23.5** | **20** | **9.8** | **0.279** | **0.024** |
| **anterior pretectal thalamic area** | **8** | **3.6** | **2** | **1.7** | **9** | **4.2** | **9** | **2.7** | **0.303** | **0.033** |
| **medial geniculate** | **5** | **2.7** | **3** | **1.5** | **7** | **3.3** | **10** | **3.6** | **0.329** | **0.015** |
| **forceps minor corpus callosum** | **2** | **0.7** | **2** | **0.7** | **5** | **1.3** | **3** | **1.0** | **0.330** | **0.016** |
| **external capsule** | **14** | **4.3** | **10** | **2.6** | **19** | **5.5** | **7** | **2.2** | **0.333** | **0.011** |
| **posterior thalamic area** | **8** | **4.1** | **2** | **2.0** | **5** | **2.7** | **6** | **1.9** | **0.411** | **-0.015** |
| **parietal ctx** | **1** | **0.5** | **3** | **1.3** | **2** | **0.8** | **3** | **1.0** | **0.412** | **0.000** |
| **dentate gyrus** | **54** | **25.8** | **16** | **7.5** | **66** | **22.5** | **20** | **6.4** | **0.454** | **-0.014** |
| **CA3** | **24** | **11.2** | **8** | **3.4** | **26** | **8.5** | **23** | **7.4** | **0.457** | **-0.013** |
| **paraventricular hypothalamic area** | **2** | **0.9** | **1** | **0.4** | **2** | **1.0** | **1** | **0.5** | **0.462** | **-0.018** |
| **anterior thalamic area** | **16** | **5.8** | **6** | **3.3** | **25** | **10.7** | **8** | **3.2** | **0.476** | **0.005** |
| **ventral thalamic area** | **41** | **21.2** | **11** | **5.3** | **50** | **17.3** | **33** | **9.7** | **0.476** | **-0.012** |
| **decussation superior cerebellar peduncle** | **1** | **0.8** | **0** | **0.3** | **0** | **0.0** | **0** | **0.0** | **0.485** | **-0.151** |
| **lateral dorsal thalamic area** | **12** | **4.9** | **3** | **1.5** | **10** | **4.6** | **7** | **3.1** | **0.514** | **-0.011** |
| **internal capsule** | **11** | **3.4** | **8** | **3.2** | **17** | **5.3** | **9** | **2.2** | **0.530** | **-0.025** |
| **retrosplenial caudal ctx** | **27** | **16.6** | **19** | **5.8** | **44** | **20.2** | **13** | **5.6** | **0.568** | **-0.014** |
| **paraventricular thalamic area** | **5** | **2.2** | **2** | **1.0** | **5** | **2.6** | **5** | **2.2** | **0.595** | **-0.028** |
| **pyramidal tracts** | **8** | **3.4** | **10** | **4.6** | **14** | **3.6** | **13** | **6.3** | **0.644** | **0.000** |
| **dorsal raphe** | **4** | **2.3** | **3** | **1.7** | **8** | **3.5** | **5** | **2.7** | **0.655** | **-0.043** |
| **lateral posterior thalamic area** | **7** | **3.6** | **2** | **1.7** | **6** | **3.4** | **5** | **2.3** | **0.661** | **-0.056** |
| **lateral geniculate** | **2** | **1.3** | **4** | **2.2** | **4** | **1.5** | **5** | **1.7** | **0.669** | **-0.045** |
| **superior colliculus** | **56** | **32.2** | **24** | **13.4** | **56** | **28.8** | **23** | **10.7** | **0.722** | **-0.047** |
| **reuniens thalamic area** | **5** | **2.9** | **5** | **3.1** | **8** | **2.9** | **3** | **1.8** | **0.732** | **-0.054** |
| **retrosplenial rostral ctx** | **37** | **12.2** | **25** | **8.7** | **39** | **13.4** | **25** | **8.2** | **0.789** | **-0.037** |
| **inferior colliculus** | **34** | **16.0** | **35** | **9.8** | **77** | **33.9** | **22** | **6.3** | **0.811** | **-0.035** |
| **zona incerta** | **3** | **1.8** | **2** | **1.1** | **5** | **2.9** | **1** | **0.9** | **0.919** | **-0.050** |

**SUPPLEMENTAL TABLE 2a** Positive Volume of Activation of NLX-112 at all doses.

| **NLX-112 Positive VOA** | | | | | | | | | | |
| --- | --- | --- | --- | --- | --- | --- | --- | --- | --- | --- |
|  | **Vehicle** | | **0.03 mg/kg** | | **0.3 mg/kg** | | **3.0 mg/kg** | |  | |
| **Brain Area** | **Mean** | **SE** | **Mean** | **SE** | **Mean** | **SE** | **Mean** | **SE** | **P va** | **Ω Sq** |
| **medullary reticular dorsal area** | **56** | **18.7** | **3** | **1.3** | **3** | **1.0** | **7** | **1.9** | **0.000** | **0.745** |
| **6th cerebellar lobule** | **3** | **1.4** | **122** | **16.2** | **4** | **2.4** | **136** | **13.0** | **0.000** | **0.620** |
| **medullary reticular ventral area** | **56** | **19.1** | **10** | **4.5** | **151** | **27.5** | **15** | **4.3** | **0.000** | **0.608** |
| **accumbens shell** | **1** | **0.6** | **30** | **4.6** | **2** | **1.3** | **23** | **3.6** | **0.000** | **0.544** |
| **tenia tecta ctx** | **4** | **2.1** | **21** | **5.8** | **25** | **5.8** | **19** | **2.8** | **0.000** | **0.526** |
| **accumbens core** | **1** | **0.9** | **19** | **4.0** | **37** | **10.5** | **19** | **3.7** | **0.001** | **0.498** |
| **anterior commissure** | **0** | **0.3** | **3** | **0.9** | **34** | **10.1** | **5** | **0.9** | **0.001** | **0.471** |
| **frontal association ctx** | **2** | **0.6** | **28** | **10.1** | **45** | **13.2** | **13** | **3.7** | **0.001** | **0.456** |
| **anterior olfactory area** | **24** | **5.3** | **101** | **16.8** | **89** | **25.4** | **39** | **7.0** | **0.001** | **0.449** |
| **8th cerebellar lobule** | **54** | **11.9** | **16** | **7.4** | **50** | **9.8** | **6** | **3.3** | **0.001** | **0.444** |
| **endopiriform area** | **0** | **0.2** | **8** | **2.0** | **29** | **8.9** | **7** | **1.9** | **0.002** | **0.430** |
| **rostral piriform ctx** | **20** | **7.4** | **78** | **17.4** | **23** | **6.1** | **31** | **7.4** | **0.002** | **0.025** |
| **3rd cerebellar lobule** | **0** | **0.0** | **24** | **6.2** | **71** | **20.0** | **14** | **2.9** | **0.003** | **0.372** |
| **pyramidal tracts** | **5** | **2.0** | **11** | **5.8** | **14** | **4.5** | **18** | **1.6** | **0.004** | **0.366** |
| **stria terminalis** | **18** | **6.0** | **1** | **0.3** | **29** | **12.1** | **0** | **0.3** | **0.004** | **0.364** |
| **10th cerebellar lobule** | **0** | **0.1** | **5** | **4.2** | **151** | **24.6** | **7** | **2.6** | **0.004** | **0.362** |
| **orbital ctx** | **11** | **3.9** | **66** | **20.5** | **53** | **18.2** | **47** | **11.3** | **0.005** | **0.350** |
| **5th cerebellar lobule** | **1** | **0.4** | **60** | **18.1** | **73** | **10.5** | **9** | **2.5** | **0.005** | **0.343** |
| **9th cerebellar lobule** | **17** | **4.6** | **9** | **4.7** | **0** | **0.0** | **5** | **1.5** | **0.005** | **0.339** |
| **diagonal band of Broca** | **3** | **1.1** | **13** | **3.8** | **220** | **22.8** | **10** | **2.9** | **0.007** | **0.319** |
| **2nd cerebellar lobule** | **13** | **3.3** | **11** | **6.0** | **32** | **15.1** | **26** | **2.8** | **0.007** | **0.317** |
| **solitary tract area** | **19** | **4.9** | **14** | **6.1** | **417** | **86.0** | **10** | **2.6** | **0.007** | **0.316** |
| **granular cell layer** | **5** | **1.5** | **59** | **16.1** | **23** | **9.6** | **28** | **6.2** | **0.009** | **0.299** |
| **posterior thalamic area** | **8** | **3.7** | **4** | **3.9** | **38** | **13.8** | **0** | **0.0** | **0.010** | **0.293** |
| **cortical amygdaloid area** | **53** | **7.7** | **62** | **7.6** | **222** | **38.3** | **31** | **4.1** | **0.010** | **0.038** |
| **lateral reticular area** | **2** | **2.3** | **6** | **2.3** | **79** | **15.6** | **5** | **1.5** | **0.011** | **0.282** |
| **ventral pallidum** | **5** | **2.5** | **31** | **8.9** | **34** | **7.4** | **24** | **6.0** | **0.012** | **0.280** |
| **anterior amygdaloid area** | **2** | **0.7** | **14** | **3.6** | **20** | **6.2** | **8** | **1.7** | **0.014** | **0.266** |
| **anterior pretectal thalamic area** | **5** | **1.7** | **5** | **3.3** | **18** | **5.3** | **1** | **0.4** | **0.015** | **0.258** |
| **corpus callosum** | **38** | **7.9** | **60** | **14.1** | **7** | **2.1** | **80** | **5.9** | **0.016** | **0.253** |
| **insular rostral ctx** | **25** | **7.7** | **60** | **11.5** | **14** | **6.0** | **31** | **6.9** | **0.017** | **0.055** |
| **locus ceruleus** | **0** | **0.0** | **1** | **0.4** | **23** | **8.4** | **0** | **0.3** | **0.019** | **0.244** |
| **habenular area** | **3** | **0.9** | **1** | **0.8** | **37** | **11.3** | **1** | **0.9** | **0.022** | **0.230** |
| **7th cerebellar lobule** | **0** | **0.1** | **24** | **7.5** | **43** | **7.2** | **19** | **6.9** | **0.027** | **0.216** |
| **ventral tegmental area** | **3** | **1.4** | **12** | **3.2** | **7** | **5.1** | **12** | **2.5** | **0.034** | **0.199** |
| **spinal trigeminal nuclear area** | **36** | **15.0** | **42** | **15.0** | **176** | **38.4** | **62** | **8.6** | **0.034** | **0.198** |
| **caudal piriform ctx** | **76** | **10.3** | **65** | **9.2** | **18** | **10.8** | **41** | **4.2** | **0.035** | **0.194** |
| **principal sensory nucleus trigeminal** | **13** | **6.3** | **21** | **7.9** | **15** | **7.2** | **33** | **3.7** | **0.037** | **0.191** |
| **insular caudal ctx** | **28** | **8.1** | **12** | **3.9** | **12** | **8.1** | **13** | **3.4** | **0.047** | **0.173** |
| **lateral rostral hypothalamic area** | **22** | **4.1** | **47** | **13.6** | **199** | **31.6** | **52** | **7.3** | **0.048** | **0.171** |
| **interpeduncular area** | **2** | **1.3** | **6** | **2.7** | **15** | **6.4** | **4** | **2.3** | **0.050** | **0.168** |
| **fimbria hippocampus** | **13** | **3.0** | **16** | **4.9** | **40** | **12.3** | **25** | **3.0** | **0.053** | **0.036** |
| **facial nucleus** | **9** | **3.5** | **2** | **0.5** | **16** | **6.6** | **6** | **1.2** | **0.060** | **0.153** |
| **medial preoptic area** | **9** | **3.1** | **19** | **6.4** | **3** | **0.9** | **24** | **5.7** | **0.061** | **0.152** |
| **lateral dorsal thalamic area** | **5** | **1.5** | **4** | **3.6** | **7** | **3.2** | **1** | **0.8** | **0.063** | **0.150** |
| **posterior hypothalamic area** | **3** | **1.4** | **5** | **1.4** | **20** | **6.3** | **2** | **0.6** | **0.065** | **0.147** |
| **bed nucleus stria terminalis** | **3** | **1.5** | **16** | **7.9** | **36** | **6.8** | **17** | **4.5** | **0.066** | **0.146** |
| **median raphe area** | **14** | **6.1** | **12** | **5.4** | **32** | **9.5** | **3** | **1.5** | **0.068** | **0.144** |
| **lateral lemniscus** | **4** | **2.6** | **5** | **3.5** | **148** | **33.3** | **11** | **1.9** | **0.068** | **0.143** |
| **medial mammillary area** | **6** | **2.8** | **10** | **5.2** | **58** | **20.7** | **5** | **3.1** | **0.071** | **0.141** |
| **ambiguus area** | **2** | **1.1** | **4** | **1.7** | **26** | **6.2** | **5** | **1.1** | **0.074** | **0.136** |
| **lateral caudal hypothalamic area** | **16** | **4.4** | **27** | **5.5** | **15** | **4.9** | **12** | **3.9** | **0.074** | **0.136** |
| **lateral posterior thalamic area** | **8** | **3.8** | **4** | **3.7** | **77** | **11.7** | **0** | **0.1** | **0.076** | **0.134** |
| **lateral amygdaloid area** | **4** | **1.8** | **5** | **2.4** | **188** | **44.1** | **1** | **0.7** | **0.078** | **0.133** |
| **olivary complex** | **7** | **3.4** | **5** | **2.9** | **23** | **5.2** | **12** | **2.5** | **0.078** | **0.133** |
| **dorsal hippocampal commissure** | **4** | **1.6** | **4** | **1.2** | **16** | **4.8** | **0** | **0.4** | **0.080** | **0.130** |
| **simple lobule cerebellum** | **24** | **11.3** | **58** | **11.6** | **11** | **4.4** | **29** | **6.6** | **0.086** | **0.125** |
| **flocculus cerebellum** | **37** | **12.0** | **50** | **8.0** | **96** | **29.1** | **62** | **6.5** | **0.093** | **0.119** |
| **pedunculopontine tegmental area** | **12** | **5.1** | **13** | **4.9** | **64** | **16.4** | **3** | **1.0** | **0.095** | **0.117** |
| **lateral preoptic area** | **2** | **0.9** | **6** | **1.9** | **23** | **4.1** | **6** | **1.4** | **0.107** | **0.030** |
| **optic tract** | **2** | **0.8** | **5** | **1.1** | **7** | **2.2** | **2** | **0.8** | **0.109** | **0.106** |
| **lateral geniculate** | **12** | **4.5** | **3** | **2.9** | **30** | **9.3** | **2** | **0.9** | **0.118** | **0.100** |
| **medial amygdaloid area** | **16** | **4.5** | **34** | **8.2** | **22** | **6.0** | **30** | **5.7** | **0.149** | **0.081** |
| **anterior thalamic area** | **5** | **2.0** | **11** | **6.0** | **1** | **0.5** | **12** | **1.2** | **0.150** | **0.080** |
| **medial dorsal thalamic area** | **5** | **1.6** | **3** | **2.6** | **2** | **0.8** | **2** | **1.0** | **0.156** | **0.077** |
| **prepositus area** | **0** | **0.1** | **3** | **2.0** | **9** | **3.5** | **0** | **0.0** | **0.168** | **0.071** |
| **substantia nigra** | **16** | **5.2** | **36** | **8.8** | **40** | **7.0** | **33** | **7.8** | **0.174** | **0.068** |
| **crus of ansiform lobule** | **118** | **33.2** | **214** | **33.5** | **2** | **1.0** | **169** | **26.5** | **0.181** | **0.065** |
| **inferior colliculus** | **89** | **26.0** | **122** | **22.0** | **42** | **9.8** | **56** | **9.4** | **0.187** | **0.062** |
| **secondary somaotsensory ctx** | **54** | **16.0** | **44** | **11.4** | **71** | **22.8** | **24** | **7.2** | **0.191** | **0.061** |
| **dorsal medial hypothalamic area** | **2** | **1.1** | **5** | **1.3** | **17** | **6.4** | **2** | **1.1** | **0.204** | **0.055** |
| **cuneate area** | **3** | **2.2** | **2** | **0.7** | **7** | **2.8** | **1** | **0.6** | **0.205** | **0.055** |
| **central medial thalamic area** | **5** | **2.3** | **3** | **1.8** | **90** | **23.6** | **7** | **2.5** | **0.231** | **0.063** |
| **lateral paragigantocellular area** | **17** | **4.6** | **19** | **6.7** | **11** | **4.5** | **24** | **4.1** | **0.250** | **0.038** |
| **decussation superior cerebellar peduncle** | **1** | **0.5** | **0** | **0.3** | **10** | **1.8** | **0** | **0.0** | **0.251** | **0.038** |
| **anterior cingulate area** | **15** | **4.3** | **26** | **12.1** | **2** | **1.0** | **22** | **4.4** | **0.252** | **0.038** |
| **cerebellar nuclear area** | **2** | **0.5** | **6** | **4.2** | **4** | **1.4** | **9** | **2.8** | **0.259** | **0.035** |
| **olfactory tubercles** | **18** | **6.4** | **21** | **4.2** | **10** | **3.7** | **13** | **3.7** | **0.264** | **0.034** |
| **central amygdaloid area** | **13** | **5.5** | **19** | **9.4** | **35** | **12.8** | **5** | **1.9** | **0.271** | **0.032** |
| **paraventricular thalamic area** | **2** | **1.1** | **4** | **1.9** | **1** | **0.6** | **7** | **2.6** | **0.273** | **0.055** |
| **paramedian lobule** | **13** | **2.9** | **42** | **13.8** | **2** | **1.0** | **20** | **6.3** | **0.280** | **0.029** |
| **pontine area** | **12** | **8.7** | **28** | **13.2** | **10** | **3.9** | **15** | **7.1** | **0.283** | **0.028** |
| **extended amydala** | **4** | **2.0** | **9** | **4.6** | **28** | **3.7** | **13** | **4.4** | **0.285** | **0.004** |
| **ventricle** | **38** | **12.5** | **54** | **16.8** | **146** | **35.3** | **65** | **10.2** | **0.287** | **0.048** |
| **temporal ctx** | **11** | **3.1** | **15** | **5.1** | **49** | **12.4** | **25** | **6.7** | **0.288** | **0.027** |
| **prelimbic ctx** | **2** | **1.5** | **6** | **5.7** | **8** | **3.1** | **8** | **4.2** | **0.306** | **0.021** |
| **reticulotegmental nucleus** | **3** | **2.2** | **5** | **4.6** | **63** | **26.3** | **0** | **0.0** | **0.307** | **0.021** |
| **retrosplenial caudal ctx** | **69** | **17.2** | **54** | **13.0** | **59** | **19.3** | **30** | **6.1** | **0.308** | **0.021** |
| **medial septal area** | **1** | **0.3** | **3** | **1.0** | **5** | **1.4** | **3** | **1.3** | **0.322** | **0.017** |
| **external capsule** | **10** | **2.6** | **13** | **5.4** | **1** | **0.5** | **10** | **2.3** | **0.346** | **0.011** |
| **pituitary** | **4** | **2.0** | **7** | **3.7** | **18** | **3.4** | **7** | **1.7** | **0.372** | **0.007** |
| **visual 1 ctx** | **199** | **32.2** | **193** | **33.6** | **2** | **1.2** | **136** | **38.6** | **0.374** | **0.004** |
| **glomerular layer** | **23** | **4.9** | **59** | **17.0** | **19** | **7.5** | **21** | **6.7** | **0.382** | **0.002** |
| **parabrachial area** | **5** | **2.2** | **6** | **2.8** | **23** | **8.0** | **1** | **0.4** | **0.385** | **0.002** |
| **pontine reticular nucleus caudal** | **18** | **8.5** | **26** | **18.9** | **7** | **2.9** | **17** | **4.4** | **0.399** | **0.002** |
| **subiculum** | **87** | **24.6** | **58** | **20.5** | **12** | **4.0** | **36** | **6.1** | **0.405** | **0.003** |
| **gigantocelllaris reticular area** | **20** | **6.7** | **49** | **26.8** | **1** | **0.5** | **37** | **5.7** | **0.408** | **0.004** |
| **superior colliculus** | **128** | **40.5** | **73** | **35.1** | **87** | **23.7** | **29** | **5.1** | **0.409** | **0.004** |
| **dorsal raphe** | **6** | **3.1** | **7** | **2.9** | **6** | **1.7** | **2** | **0.6** | **0.412** | **0.005** |
| **secondary motor ctx** | **31** | **11.0** | **35** | **25.4** | **12** | **3.6** | **31** | **7.4** | **0.435** | **0.058** |
| **caudate putamen** | **97** | **20.6** | **142** | **80.9** | **1** | **0.7** | **76** | **25.3** | **0.462** | **0.015** |
| **internal capsule** | **17** | **5.1** | **15** | **6.3** | **389** | **84.7** | **9** | **3.1** | **0.480** | **0.018** |
| **globus pallidus** | **16** | **6.7** | **23** | **12.4** | **106** | **27.7** | **8** | **3.4** | **0.485** | **0.019** |
| **infralimbic ctx** | **1** | **0.3** | **3** | **1.1** | **8** | **3.1** | **1** | **0.5** | **0.573** | **0.035** |
| **primary somatosensory ctx** | **172** | **40.1** | **182** | **64.8** | **10** | **2.6** | **114** | **26.4** | **0.585** | **0.037** |
| **fornix** | **0** | **0.1** | **1** | **0.3** | **104** | **29.1** | **1** | **0.3** | **0.599** | **0.039** |
| **parietal ctx** | **2** | **1.4** | **3** | **0.9** | **98** | **24.2** | **4** | **1.2** | **0.601** | **0.028** |
| **anterior hypothalamic area** | **28** | **7.8** | **39** | **10.7** | **75** | **26.1** | **25** | **7.5** | **0.605** | **0.036** |
| **reuniens thalamic area** | **4** | **1.9** | **6** | **2.0** | **86** | **30.7** | **8** | **3.1** | **0.610** | **0.057** |
| **parafascicular thalamic area** | **1** | **0.4** | **1** | **0.5** | **64** | **23.5** | **0** | **0.1** | **0.617** | **0.042** |
| **periaqueductal gray** | **56** | **18.8** | **65** | **19.9** | **9** | **4.2** | **32** | **4.4** | **0.627** | **0.043** |
| **dentate gyrus** | **87** | **28.6** | **51** | **18.5** | **7** | **3.3** | **47** | **7.2** | **0.644** | **0.046** |
| **ventral thalamic area** | **36** | **18.4** | **41** | **22.5** | **5** | **1.3** | **40** | **8.8** | **0.646** | **0.046** |
| **basal amygdaloid area** | **29** | **5.5** | **36** | **11.4** | **19** | **5.5** | **30** | **2.9** | **0.652** | **0.047** |
| **paraventricular hypothalamic area** | **2** | **1.0** | **1** | **0.6** | **5** | **2.7** | **1** | **0.4** | **0.655** | **0.048** |
| **lateral septal area** | **12** | **2.7** | **18** | **12.0** | **2** | **0.8** | **19** | **7.0** | **0.655** | **0.048** |
| **cerebral peduncle** | **31** | **12.1** | **40** | **17.2** | **66** | **22.5** | **37** | **5.7** | **0.704** | **0.055** |
| **forceps minor corpus callosum** | **2** | **0.6** | **2** | **1.2** | **26** | **8.5** | **2** | **0.7** | **0.720** | **0.057** |
| **primary motor ctx** | **30** | **11.5** | **42** | **17.0** | **2** | **1.0** | **21** | **2.3** | **0.747** | **0.004** |
| **auditory ctx** | **35** | **6.3** | **27** | **6.4** | **25** | **10.7** | **48** | **13.6** | **0.747** | **0.061** |
| **parvicellular reticular area** | **15** | **4.8** | **13** | **5.4** | **50** | **17.3** | **13** | **1.3** | **0.784** | **0.067** |
| **reticular thalamic area** | **9** | **4.3** | **8** | **4.9** | **0** | **0.0** | **6** | **2.0** | **0.796** | **0.068** |
| **vestibular area** | **10** | **4.6** | **20** | **11.2** | **10** | **4.6** | **11** | **4.9** | **0.845** | **0.075** |
| **CA1** | **81** | **28.1** | **78** | **23.1** | **17** | **5.3** | **89** | **12.3** | **0.873** | **0.079** |
| **lemniscal area** | **6** | **3.4** | **6** | **3.3** | **44** | **20.2** | **3** | **1.4** | **0.879** | **0.080** |
| **medial geniculate** | **20** | **5.8** | **15** | **6.2** | **5** | **2.6** | **15** | **3.5** | **0.883** | **0.081** |
| **CA3** | **34** | **11.4** | **35** | **10.0** | **14** | **3.6** | **36** | **5.0** | **0.892** | **0.082** |
| **zona incerta** | **10** | **2.8** | **12** | **4.5** | **8** | **3.5** | **15** | **4.0** | **0.906** | **0.018** |
| **intermediate reticular area** | **16** | **4.9** | **21** | **7.5** | **6** | **3.4** | **20** | **4.5** | **0.910** | **0.085** |
| **entorhinal ctx** | **199** | **32.3** | **213** | **30.3** | **4** | **1.5** | **210** | **14.4** | **0.919** | **0.086** |
| **pontine reticular nucleus oral** | **30** | **12.6** | **26** | **17.0** | **56** | **28.8** | **14** | **2.3** | **0.939** | **0.090** |
| **4th cerebellar lobule** | **9** | **3.6** | **6** | **4.1** | **8** | **2.9** | **5** | **1.7** | **0.947** | **0.091** |
| **mesencephalic reticular formation** | **71** | **23.9** | **65** | **30.8** | **39** | **13.4** | **51** | **12.8** | **0.968** | **0.095** |
| **retrosplenial rostral ctx** | **42** | **14.5** | **40** | **14.5** | **77** | **33.9** | **38** | **5.5** | **0.976** | **0.045** |
| **ventral medial hypothalamic area** | **9** | **3.9** | **7** | **2.5** | **5** | **2.9** | **7** | **2.5** | **0.996** | **0.065** |

**SUPPLEMENTAL TABLE 2b** Negative Volume of Activation of NLX-112 at all doses.

| **NLX-112 Negative VOA** | | | | | | | | | | |
| --- | --- | --- | --- | --- | --- | --- | --- | --- | --- | --- |
|  | **Vehicle** | | **0.03 mg/kg** | | **0.3 mg/kg** | | **3.0 mg/kg** | |  | |
| **Brain Area** | **Mean** | **SE** | **Mean** | **SE** | **Mean** | **SE** | **Mean** | **SE** | **P va** | **Ω Sq** |
| **medullary reticular dorsal area** | **38** | **11.8** | **3** | **1.6** | **0** | **0.0** | **6** | **1.3** | **0.000** | **0.737** |
| **ambiguus area** | **70** | **23.6** | **3** | **0.9** | **2** | **0.6** | **7** | **1.2** | **0.000** | **0.546** |
| **prepositus area** | **2** | **0.9** | **3** | **1.1** | **4** | **1.6** | **16** | **1.1** | **0.000** | **0.562** |
| **gigantocelllaris reticular area** | **16** | **4.9** | **53** | **15.5** | **75** | **19.8** | **147** | **16.5** | **0.001** | **0.458** |
| **5th cerebellar lobule** | **3** | **1.3** | **12** | **9.9** | **16** | **6.7** | **64** | **11.7** | **0.001** | **0.487** |
| **interpeduncular area** | **5** | **2.5** | **1** | **1.0** | **2** | **1.3** | **15** | **2.6** | **0.001** | **0.468** |
| **reticulotegmental nucleus** | **13** | **5.6** | **2** | **0.9** | **12** | **4.9** | **35** | **2.3** | **0.001** | **0.475** |
| **lateral reticular area** | **31** | **7.4** | **7** | **3.1** | **0** | **0.0** | **8** | **2.9** | **0.001** | **0.456** |
| **pontine reticular nucleus caudal** | **35** | **14.8** | **29** | **10.0** | **43** | **13.7** | **130** | **10.7** | **0.001** | **0.406** |
| **pedunculopontine tegmental area** | **12** | **6.0** | **8** | **2.2** | **10** | **2.7** | **37** | **4.7** | **0.002** | **0.375** |
| **parabrachial area** | **4** | **1.9** | **2** | **1.1** | **7** | **2.5** | **17** | **1.9** | **0.002** | **0.368** |
| **medullary reticular ventral area** | **37** | **18.0** | **7** | **5.0** | **0** | **0.0** | **25** | **5.1** | **0.002** | **0.423** |
| **intermediate reticular area** | **7** | **3.7** | **20** | **6.6** | **22** | **5.9** | **49** | **6.8** | **0.002** | **0.348** |
| **cerebral peduncle** | **32** | **12.5** | **20** | **9.9** | **47** | **13.3** | **103** | **13.4** | **0.003** | **0.333** |
| **lateral geniculate** | **2** | **1.3** | **2** | **0.9** | **9** | **2.4** | **15** | **3.4** | **0.004** | **0.345** |
| **median raphe area** | **16** | **6.9** | **6** | **2.1** | **12** | **5.2** | **39** | **3.6** | **0.004** | **0.331** |
| **superior colliculus** | **56** | **32.2** | **28** | **9.3** | **53** | **18.0** | **140** | **15.6** | **0.005** | **0.313** |
| **spinal trigeminal nuclear area** | **12** | **4.6** | **44** | **8.4** | **50** | **12.1** | **72** | **10.5** | **0.005** | **0.328** |
| **paramedian lobule** | **10** | **4.1** | **24** | **7.7** | **34** | **9.2** | **69** | **13.6** | **0.005** | **0.309** |
| **pontine reticular nucleus oral** | **42** | **19.4** | **8** | **3.5** | **33** | **15.6** | **87** | **10.5** | **0.005** | **0.296** |
| **posterior thalamic area** | **8** | **4.1** | **3** | **1.8** | **6** | **2.9** | **22** | **3.5** | **0.005** | **0.347** |
| **posterior hypothalamic area** | **2** | **0.9** | **1** | **0.6** | **1** | **0.6** | **7** | **1.2** | **0.006** | **0.303** |
| **vestibular area** | **12** | **7.2** | **17** | **6.4** | **29** | **9.1** | **78** | **14.1** | **0.007** | **0.282** |
| **lateral posterior thalamic area** | **7** | **3.6** | **7** | **2.5** | **11** | **3.9** | **21** | **1.8** | **0.007** | **0.315** |
| **subiculum** | **38** | **27.0** | **20** | **11.8** | **60** | **16.2** | **78** | **11.0** | **0.008** | **0.275** |
| **locus ceruleus** | **3** | **2.1** | **0** | **0.3** | **1** | **0.4** | **2** | **0.3** | **0.009** | **0.301** |
| **anterior pretectal thalamic area** | **8** | **3.6** | **6** | **2.3** | **9** | **2.4** | **20** | **2.3** | **0.011** | **0.286** |
| **cuneate area** | **1** | **0.6** | **1** | **0.6** | **0** | **0.0** | **4** | **0.8** | **0.012** | **0.272** |
| **olivary complex** | **4** | **2.0** | **2** | **0.8** | **6** | **2.0** | **13** | **2.9** | **0.012** | **0.266** |
| **dentate gyrus** | **54** | **25.8** | **47** | **14.5** | **71** | **13.2** | **125** | **10.9** | **0.013** | **0.238** |
| **7th cerebellar lobule** | **2** | **0.9** | **9** | **8.0** | **5** | **2.6** | **31** | **13.6** | **0.014** | **0.270** |
| **4th cerebellar lobule** | **6** | **3.4** | **1** | **1.1** | **7** | **3.5** | **15** | **4.7** | **0.015** | **0.226** |
| **glomerular layer** | **162** | **26.4** | **50** | **13.0** | **82** | **21.6** | **63** | **14.1** | **0.015** | **0.232** |
| **parvicellular reticular area** | **14** | **3.8** | **18** | **3.0** | **21** | **6.2** | **36** | **3.1** | **0.016** | **0.224** |
| **8th cerebellar lobule** | **56** | **19.5** | **9** | **6.7** | **5** | **3.3** | **46** | **15.4** | **0.016** | **0.277** |
| **temporal ctx** | **24** | **4.8** | **10** | **4.3** | **14** | **3.2** | **6** | **2.8** | **0.018** | **0.223** |
| **cerebellar nuclear area** | **3** | **1.1** | **5** | **2.8** | **12** | **3.9** | **23** | **5.4** | **0.019** | **0.221** |
| **endopiriform area** | **15** | **3.2** | **5** | **2.1** | **6** | **1.5** | **8** | **1.5** | **0.019** | **0.261** |
| **10th cerebellar lobule** | **2** | **0.9** | **5** | **2.3** | **11** | **4.4** | **16** | **3.4** | **0.020** | **0.267** |
| **stria terminalis** | **6** | **4.7** | **0** | **0.1** | **0** | **0.0** | **0** | **0.2** | **0.020** | **0.242** |
| **retrosplenial caudal ctx** | **27** | **16.6** | **15** | **8.4** | **22** | **6.0** | **54** | **11.9** | **0.020** | **0.216** |
| **principal sensory nucleus trigeminal** | **29** | **12.2** | **11** | **4.7** | **26** | **9.4** | **46** | **6.0** | **0.021** | **0.230** |
| **pontine area** | **28** | **12.6** | **23** | **11.4** | **20** | **9.9** | **72** | **8.7** | **0.021** | **0.214** |
| **lateral septal area** | **48** | **8.0** | **13** | **6.9** | **20** | **4.6** | **30** | **9.2** | **0.021** | **0.205** |
| **accumbens core** | **18** | **4.2** | **3** | **1.3** | **4** | **1.6** | **11** | **2.5** | **0.024** | **0.253** |
| **CA1** | **67** | **29.8** | **62** | **16.8** | **95** | **11.3** | **134** | **10.9** | **0.028** | **0.183** |
| **simple lobule cerebellum** | **26** | **7.9** | **11** | **6.2** | **35** | **7.7** | **39** | **7.6** | **0.028** | **0.202** |
| **lateral rostral hypothalamic area** | **14** | **4.7** | **20** | **8.7** | **19** | **4.0** | **37** | **5.5** | **0.029** | **0.207** |
| **caudate putamen** | **286** | **38.8** | **127** | **66.5** | **176** | **49.4** | **317** | **60.7** | **0.029** | **0.187** |
| **secondary somaotsensory ctx** | **46** | **17.4** | **35** | **9.6** | **44** | **11.9** | **86** | **9.3** | **0.030** | **0.192** |
| **mesencephalic reticular formation** | **58** | **32.5** | **8** | **2.7** | **35** | **12.7** | **72** | **17.9** | **0.030** | **0.175** |
| **lateral paragigantocellular area** | **5** | **1.7** | **9** | **2.6** | **18** | **3.8** | **15** | **2.8** | **0.032** | **0.185** |
| **periaqueductal gray** | **64** | **24.6** | **23** | **15.1** | **39** | **9.0** | **74** | **8.6** | **0.036** | **0.169** |
| **lateral caudal hypothalamic area** | **7** | **2.8** | **8** | **4.1** | **4** | **1.2** | **19** | **3.7** | **0.037** | **0.185** |
| **inferior colliculus** | **34** | **16.0** | **10** | **4.8** | **38** | **10.4** | **58** | **13.4** | **0.038** | **0.176** |
| **infralimbic ctx** | **3** | **0.9** | **1** | **0.5** | **1** | **0.5** | **1** | **0.8** | **0.039** | **0.163** |
| **9th cerebellar lobule** | **5** | **1.7** | **13** | **5.4** | **8** | **5.2** | **36** | **8.7** | **0.041** | **0.206** |
| **dorsal medial hypothalamic area** | **4** | **1.5** | **4** | **1.3** | **2** | **0.7** | **9** | **1.5** | **0.041** | **0.179** |
| **primary somatosensory ctx** | **185** | **46.4** | **126** | **53.6** | **163** | **37.2** | **325** | **66.8** | **0.047** | **0.152** |
| **parafascicular thalamic area** | **2** | **0.6** | **0** | **0.1** | **1** | **0.7** | **3** | **1.1** | **0.047** | **0.183** |
| **lateral lemniscus** | **8** | **3.9** | **2** | **0.9** | **6** | **2.9** | **9** | **1.5** | **0.047** | **0.169** |
| **substantia nigra** | **5** | **2.8** | **3** | **1.9** | **6** | **1.6** | **12** | **3.1** | **0.048** | **0.158** |
| **central amygdaloid area** | **29** | **8.5** | **10** | **4.7** | **16** | **4.3** | **27** | **4.2** | **0.051** | **0.153** |
| **medial septal area** | **4** | **0.9** | **2** | **1.0** | **1** | **0.9** | **2** | **1.0** | **0.068** | **0.128** |
| **granular cell layer** | **113** | **21.0** | **45** | **13.6** | **75** | **18.0** | **69** | **12.4** | **0.081** | **0.147** |
| **medial mammillary area** | **9** | **4.5** | **14** | **5.7** | **5** | **1.9** | **22** | **4.2** | **0.082** | **0.131** |
| **visual 1 ctx** | **141** | **33.4** | **54** | **25.7** | **108** | **16.5** | **108** | **37.1** | **0.082** | **0.116** |
| **auditory ctx** | **41** | **7.6** | **13** | **7.2** | **24** | **6.1** | **41** | **13.9** | **0.085** | **0.107** |
| **fornix** | **1** | **0.4** | **0** | **0.3** | **1** | **0.4** | **1** | **0.4** | **0.087** | **0.120** |
| **solitary tract area** | **23** | **9.0** | **21** | **4.9** | **22** | **5.6** | **40** | **6.0** | **0.088** | **0.144** |
| **anterior commissure** | **4** | **1.2** | **1** | **0.4** | **1** | **0.4** | **2** | **1.2** | **0.091** | **0.148** |
| **ventral pallidum** | **14** | **5.1** | **18** | **7.7** | **14** | **4.3** | **32** | **5.4** | **0.092** | **0.136** |
| **CA3** | **24** | **11.2** | **31** | **7.0** | **39** | **4.0** | **54** | **8.0** | **0.098** | **0.095** |
| **2nd cerebellar lobule** | **2** | **1.1** | **14** | **8.0** | **22** | **9.2** | **23** | **5.5** | **0.109** | **0.126** |
| **internal capsule** | **11** | **3.4** | **7** | **5.6** | **14** | **5.8** | **23** | **7.0** | **0.110** | **0.092** |
| **reticular thalamic area** | **7** | **2.4** | **4** | **3.7** | **10** | **3.8** | **13** | **5.0** | **0.113** | **0.088** |
| **pyramidal tracts** | **8** | **3.4** | **4** | **1.8** | **9** | **3.7** | **15** | **3.8** | **0.119** | **0.126** |
| **lateral dorsal thalamic area** | **12** | **4.9** | **11** | **3.0** | **14** | **3.6** | **21** | **1.5** | **0.120** | **0.114** |
| **lemniscal area** | **7** | **4.0** | **4** | **2.1** | **6** | **2.0** | **13** | **3.4** | **0.125** | **0.079** |
| **anterior olfactory area** | **102** | **16.0** | **50** | **16.8** | **75** | **14.8** | **80** | **15.8** | **0.129** | **0.128** |
| **3rd cerebellar lobule** | **2** | **1.2** | **3** | **2.7** | **4** | **1.8** | **14** | **5.6** | **0.129** | **0.115** |
| **facial nucleus** | **20** | **7.2** | **1** | **0.5** | **4** | **1.6** | **4** | **1.6** | **0.132** | **0.093** |
| **entorhinal ctx** | **146** | **29.4** | **116** | **37.1** | **187** | **32.6** | **190** | **21.9** | **0.141** | **0.068** |
| **dorsal hippocampal commissure** | **2** | **1.1** | **0** | **0.0** | **2** | **1.3** | **2** | **1.1** | **0.146** | **0.067** |
| **bed nucleus stria terminalis** | **16** | **5.2** | **7** | **5.0** | **9** | **4.1** | **12** | **4.4** | **0.148** | **0.087** |
| **insular caudal ctx** | **24** | **8.3** | **30** | **5.5** | **26** | **4.5** | **40** | **6.0** | **0.153** | **0.088** |
| **olfactory tubercles** | **14** | **4.9** | **23** | **5.0** | **21** | **4.1** | **10** | **2.5** | **0.161** | **0.071** |
| **optic tract** | **2** | **0.9** | **1** | **0.6** | **0** | **0.3** | **2** | **0.7** | **0.161** | **0.059** |
| **globus pallidus** | **21** | **6.0** | **12** | **10.3** | **24** | **11.0** | **31** | **12.8** | **0.192** | **0.052** |
| **frontal association ctx** | **62** | **10.3** | **35** | **8.1** | **33** | **5.4** | **42** | **10.5** | **0.192** | **0.099** |
| **flocculus cerebellum** | **34** | **11.7** | **31** | **5.8** | **46** | **7.2** | **49** | **5.8** | **0.197** | **0.064** |
| **prelimbic ctx** | **23** | **6.1** | **13** | **7.8** | **8** | **4.5** | **15** | **8.0** | **0.210** | **0.056** |
| **crus of ansiform lobule** | **77** | **18.7** | **79** | **24.0** | **99** | **15.0** | **134** | **28.4** | **0.212** | **0.054** |
| **caudal piriform ctx** | **48** | **8.2** | **58** | **9.0** | **67** | **14.7** | **78** | **11.1** | **0.218** | **0.064** |
| **orbital ctx** | **104** | **22.4** | **50** | **11.6** | **52** | **8.2** | **75** | **18.4** | **0.227** | **0.076** |
| **anterior cingulate area** | **40** | **9.5** | **26** | **14.1** | **28** | **6.7** | **23** | **5.7** | **0.231** | **0.044** |
| **external capsule** | **14** | **4.3** | **10** | **5.2** | **13** | **3.2** | **22** | **5.9** | **0.233** | **0.042** |
| **habenular area** | **4** | **0.9** | **4** | **1.1** | **3** | **1.1** | **6** | **1.1** | **0.243** | **0.071** |
| **accumbens shell** | **15** | **3.6** | **9** | **4.1** | **8** | **2.9** | **14** | **2.6** | **0.245** | **0.089** |
| **forceps minor corpus callosum** | **2** | **0.7** | **1** | **0.8** | **3** | **1.0** | **1** | **0.5** | **0.250** | **0.018** |
| **ventral thalamic area** | **41** | **21.2** | **19** | **18.3** | **38** | **18.4** | **56** | **24.5** | **0.258** | **0.026** |
| **medial geniculate** | **5** | **2.7** | **2** | **0.9** | **6** | **2.8** | **13** | **5.6** | **0.262** | **0.014** |
| **medial preoptic area** | **17** | **6.2** | **22** | **5.2** | **12** | **4.5** | **23** | **4.1** | **0.287** | **0.039** |
| **decussation superior cerebellar peduncle** | **1** | **0.8** | **0** | **0.0** | **0** | **0.0** | **0** | **0.2** | **0.290** | **0.027** |
| **paraventricular hypothalamic area** | **2** | **0.9** | **1** | **0.5** | **2** | **0.7** | **3** | **0.8** | **0.293** | **0.045** |
| **dorsal raphe** | **4** | **2.3** | **3** | **1.0** | **7** | **2.8** | **9** | **3.2** | **0.300** | **0.021** |
| **anterior amygdaloid area** | **5** | **2.2** | **5** | **2.7** | **8** | **2.8** | **10** | **2.1** | **0.301** | **0.045** |
| **corpus callosum** | **49** | **11.3** | **35** | **17.0** | **47** | **8.2** | **37** | **8.8** | **0.320** | **0.040** |
| **basal amygdaloid area** | **40** | **6.6** | **34** | **8.1** | **46** | **10.3** | **53** | **5.4** | **0.321** | **0.011** |
| **anterior thalamic area** | **16** | **5.8** | **12** | **5.5** | **19** | **3.5** | **17** | **3.4** | **0.338** | **0.021** |
| **ventral tegmental area** | **3** | **1.8** | **2** | **1.2** | **2** | **0.9** | **3** | **1.1** | **0.341** | **0.020** |
| **extended amydala** | **6** | **3.0** | **3** | **1.9** | **3** | **1.7** | **6** | **2.1** | **0.365** | **-0.004** |
| **zona incerta** | **3** | **1.8** | **1** | **0.4** | **4** | **2.3** | **4** | **2.5** | **0.375** | **-0.018** |
| **primary motor ctx** | **68** | **18.5** | **51** | **17.4** | **62** | **11.9** | **90** | **17.8** | **0.382** | **-0.004** |
| **medial amygdaloid area** | **28** | **7.5** | **17** | **5.7** | **24** | **6.1** | **26** | **4.4** | **0.389** | **0.008** |
| **tenia tecta ctx** | **18** | **4.7** | **11** | **3.9** | **7** | **2.0** | **9** | **2.0** | **0.391** | **0.051** |
| **lateral amygdaloid area** | **8** | **3.0** | **3** | **1.0** | **7** | **2.3** | **7** | **1.8** | **0.417** | **0.010** |
| **pituitary** | **10** | **5.3** | **18** | **4.7** | **12** | **3.3** | **12** | **1.6** | **0.434** | **-0.007** |
| **parietal ctx** | **1** | **0.5** | **2** | **1.6** | **2** | **0.9** | **0** | **0.2** | **0.442** | **-0.028** |
| **diagonal band of Broca** | **6** | **2.8** | **7** | **3.3** | **5** | **1.9** | **9** | **1.4** | **0.466** | **0.016** |
| **medial dorsal thalamic area** | **8** | **2.4** | **5** | **2.3** | **8** | **3.3** | **10** | **2.3** | **0.485** | **0.005** |
| **anterior hypothalamic area** | **29** | **8.6** | **31** | **10.8** | **27** | **6.9** | **42** | **7.2** | **0.577** | **-0.036** |
| **retrosplenial rostral ctx** | **37** | **12.2** | **38** | **17.6** | **30** | **6.9** | **42** | **7.2** | **0.600** | **-0.045** |
| **lateral preoptic area** | **2** | **1.4** | **5** | **2.4** | **2** | **0.9** | **3** | **1.6** | **0.608** | **-0.030** |
| **ventricle** | **95** | **19.2** | **70** | **20.9** | **76** | **17.7** | **96** | **12.7** | **0.707** | **-0.048** |
| **reuniens thalamic area** | **5** | **2.9** | **4** | **2.9** | **6** | **2.4** | **5** | **2.1** | **0.716** | **-0.057** |
| **fimbria hippocampus** | **25** | **6.2** | **24** | **7.2** | **30** | **5.2** | **32** | **3.2** | **0.720** | **-0.036** |
| **ventral medial hypothalamic area** | **10** | **3.6** | **12** | **2.7** | **11** | **3.4** | **8** | **2.3** | **0.743** | **-0.065** |
| **6th cerebellar lobule** | **23** | **10.0** | **28** | **11.8** | **36** | **16.9** | **28** | **5.9** | **0.749** | **0.003** |
| **secondary motor ctx** | **62** | **19.2** | **50** | **23.8** | **53** | **7.9** | **51** | **13.7** | **0.762** | **-0.058** |
| **paraventricular thalamic area** | **5** | **2.2** | **4** | **2.5** | **4** | **1.4** | **3** | **1.2** | **0.801** | **-0.055** |
| **central medial thalamic area** | **6** | **2.5** | **3** | **2.1** | **2** | **0.8** | **2** | **1.0** | **0.827** | **-0.063** |
| **rostral piriform ctx** | **93** | **27.7** | **73** | **15.8** | **89** | **20.4** | **88** | **14.3** | **0.827** | **-0.025** |
| **cortical amygdaloid area** | **42** | **10.5** | **31** | **4.6** | **42** | **8.2** | **32** | **4.8** | **0.828** | **-0.038** |
| **insular rostral ctx** | **70** | **20.3** | **65** | **12.5** | **66** | **12.2** | **73** | **5.5** | **0.922** | **-0.055** |
